# Secondary Metabolites from Rubiaceae Species

**DOI:** 10.3390/molecules200713422

**Published:** 2015-07-22

**Authors:** Daiane Martins, Cecilia Veronica Nunez

**Affiliations:** Bioprospection and Biotechnology Laboratory, Technology and Innovation Coordenation, National Research Institute of Amazonia, Av. André Araújo, 2936, Petrópolis, Manaus, AM 69067-375, Brazil

**Keywords:** Rubiaceae, Rubioideae, Cinchonoideae, Ixoroideae, iridoids, alkaloid, anthraquinones, triterpenes

## Abstract

This study describes some characteristics of the Rubiaceae family pertaining to the occurrence and distribution of secondary metabolites in the main genera of this family. It reports the review of phytochemical studies addressing all species of Rubiaceae, published between 1990 and 2014. Iridoids, anthraquinones, triterpenes, indole alkaloids as well as other varying alkaloid subclasses, have shown to be the most common. These compounds have been mostly isolated from the genera *Uncaria*, *Psychotria*, *Hedyotis*, *Ophiorrhiza* and *Morinda*. The occurrence and distribution of iridoids, alkaloids and anthraquinones point out their chemotaxonomic correlation among tribes and subfamilies. From an evolutionary point of view, Rubioideae is the most ancient subfamily, followed by Ixoroideae and finally Cinchonoideae. The chemical biosynthetic pathway, which is not so specific in Rubioideae, can explain this and large amounts of both iridoids and indole alkaloids are produced. In Ixoroideae, the most active biosysthetic pathway is the one that produces iridoids; while in Cinchonoideae, it produces indole alkaloids together with other alkaloids. The chemical biosynthetic pathway now supports this botanical conclusion.

## 1. Introduction

The Rubiaceae family is characterized by the production of bioactive metabolites with great pharmacological potential. These metabolites can be used as chemotaxonomic markers even for genera and subfamilies [1,2]. Usually, taxa are classified according to different botanical characteristics; classical taxonomic systems only consider the plant morphological characters, while modern systems correlate their various combinations, including the chemical composition. Studies correlating classical plant taxonomy to chemical data can be found as far back as 1699 [3].

Phytochemical compounds can be a useful tool for characterizing, describing and classifying plant species. The distribution of secondary metabolites in Rubiaceae follows patterns that may help characterize the botanical group (subfamily, tribe or genera). These patterns relative to chemotaxonomy are often used to establish the botanical origin [4].

In recent years, Rubiaceae species have been thoroughly studied from a phytochemical viewpoint. However, very few studies have used this knowledge as a tool in taxonomic studies. When conducting bioprospecting studies of a plant, all botanical and chemotaxonomic information is of great importance, since it increases the likelihood of finding bioactive compounds, which enables the discovery of new Nature-originated drugs [5]. Therefore, the present study aims to conduct a literature survey on phytochemical studies addressing species of Rubiaceae published from 1990 to 2014, and describe their secondary metabolites occurrence and distribution in the subfamilies, tribes and main genera of this family.

## 2. Taxonomic Classification of Rubiaceae

The Rubiaceae family has a cosmopolitan distribution, mostly concentrated in the tropics. Being one of the largest in the Magnoliopsida class, it ranks fourth in diversity of species among Angiosperms [4]. It includes approximately 637 genera and 13,000 species [5,6]. In Brazil, nearly 120 genera and 1400 species occur, representing one of the most important economic, ornamental and medicinal plant families in the Brazilian flora [7].

**Figure 1:**
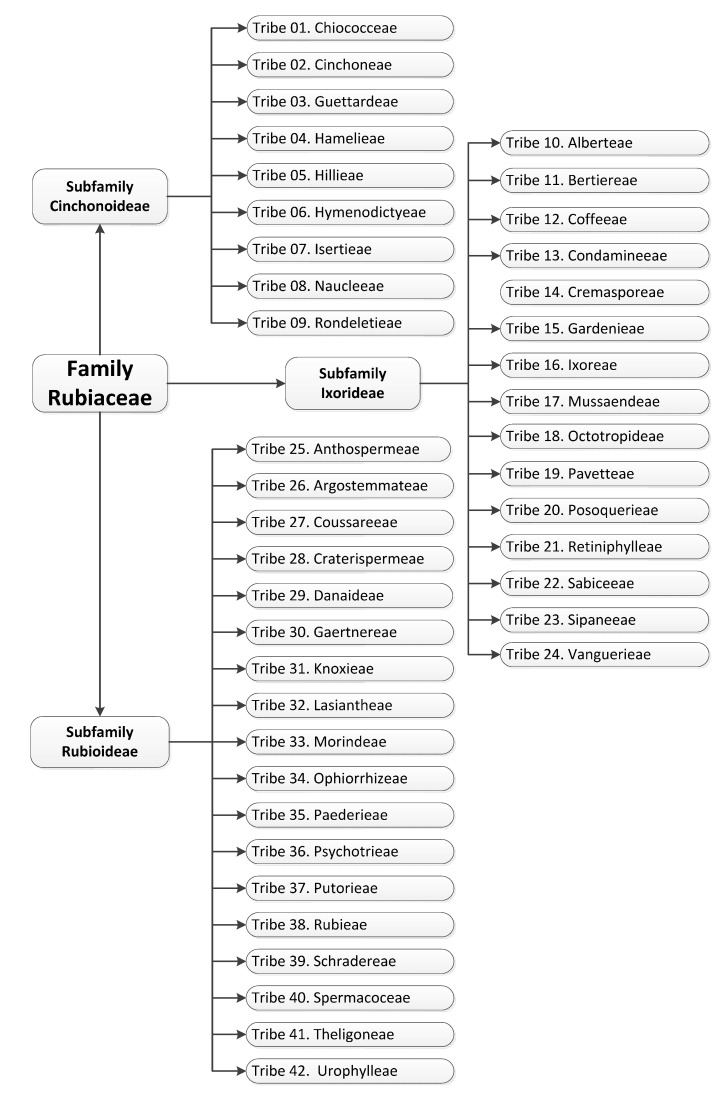
Subfamilies and tribes belonging to the Rubiaceae family [16].

The Rubiaceae family taxonomic classification is complex and there are still some gaps which have to be filled. According to the classification of Robbrecht [8], the Rubiaceae family is divided into four subfamilies: Rubioideae, Cinchonoideae, Antirheoideae and Ixoroideae. However, more recent studies suggest this family to be divided into three subfamilies: Rubioideae, Cinchonoideae and Ixoroideae, as some authors do not recognize Antirheoideae as a subfamily, since molecular studies have shown it to be polyphyletic with no standardized occurrence of a chemical marker [9,10,11,12,13,14,15,16]. Due to the abundance of species, the subfamilies were divided into 43 tribes (an intermediate clade between genus and subfamily) [16], which are listed in Figure 1.

Due to the lack of studies that can complement the extant information on geographical distribution, morpho-anatomical characteristics and molecular data, there are still genera and species not allocated into any tribe [16]. The evaluation of the chemical profile of these species may indicate a more complete phylogenetic distribution, since the secondary metabolites are the results of adaptation and evolution of a specific taxon to environment [17]. Thus, the profile of secondary metabolites distribution can bring new information for the taxonomic classification of this family.

## 3. Chemical and Biological Aspects of Rubiaceae

The Rubiaceae family presents a large diversity of substances such as iridoids, indole alkaloids, anthraquinones, terpenoids (diterpenes and triterpenes), flavonoids and other phenolic derivatives, with emphasis on production of bioactive alkaloids [2]. Alkaloids are secondary metabolites that can generate various drugs with important pharmacological effects and used to find out physiological responses and biochemical mechanisms of action [18].

The number of described products, the structural diversity and pharmacological activities reported for various species of Rubiaceae demonstrate this family to be a promising source of new bioactive substances, which may give rise to new products as active molecules or even drug prototypes. Many of these plants have widespread use in folk medicine and some showed anti-inflammatory, analgesic, antibacterial, mutagenic, antiviral, antioxidant, effect on vascular diseases as well as activity on the central nervous system [19].

In the Ixoroideae subfamily, the genus *Coffea* is one of the most economically important, mainly the species *Coffea arabica*, popularly known as coffee, which has caffeine as one of its principal chemical components. This substance acts as stimulant of the central nervous system, as well as vasoconstrictor, bronchodilator and diuretic, besides being one of the components of migraine drugs [18]. *Genipa*, the Brazilian jenipapo (*Genipa americana*) with antiangiogenic, anti-inflammatory and antioxidant activity [20,21,22] is another important genus from which genipin was isolated, a colorless iridoid, used by indigenous people to tattoo their skin, since it produces a black coloration when it reacts with skin proteins. Its fruits are used to make wines, liqueurs, jams, soft drinks, *etc.* [23].

In the Cinchonoideae subfamily, *Cinchona* species are the source of quinine, isolated in 1820 by Pelletier and Caventou [24], and which for about 200 years was the only active substance against malaria, and can be considered as responsible for the development of synthetic antimalarials [1,25]. More than 50 new substances were isolated from alkaloid-rich *Uncaria* species [19], as *Uncaria tomentosa*, known as “unha de gato”, is one of most used plants in Brazilian folk medicine. Studies have shown that alkaloids isolated from this plant have immunostimulant and antitumor activity [26,27]. Other groups of substances such as triterpenes and procyanidins presented anti-inflammatory activity [28,29].

**Figure 2:**
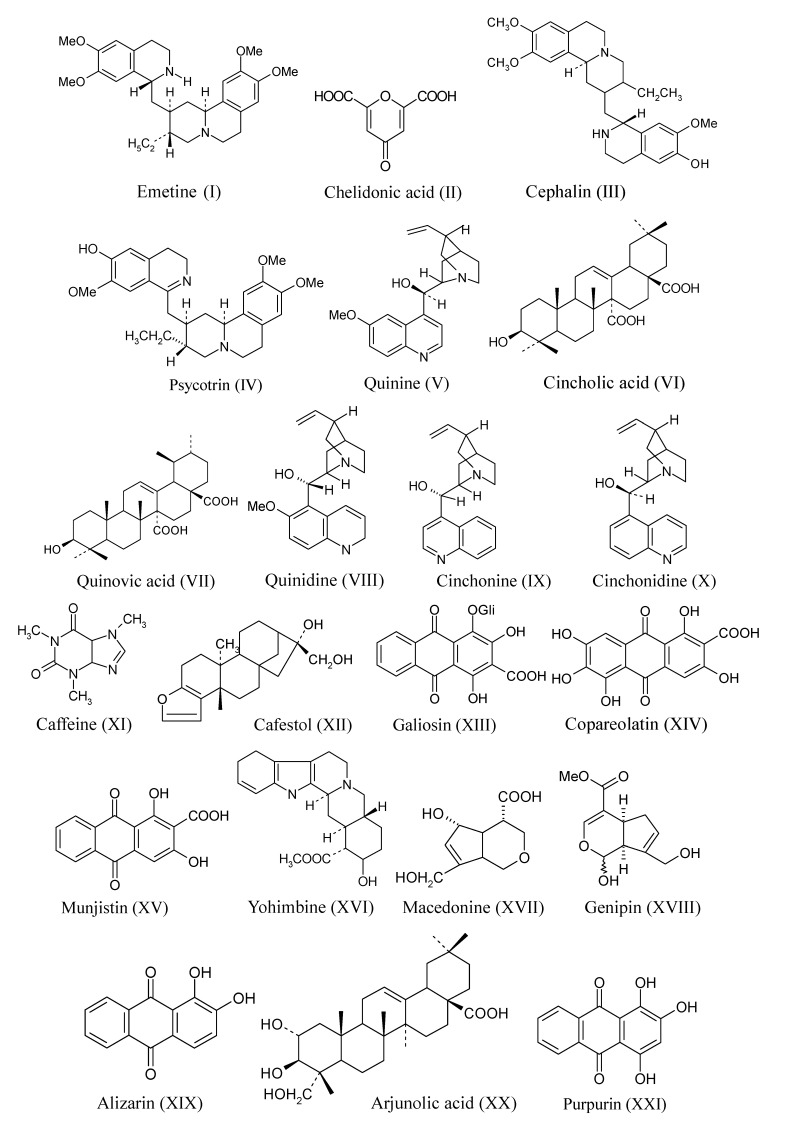
Different classes of compounds isolated from Rubiaceae.

*Psychotria*, belonging to the Rubioideae subfamily, are plants that produce substances with activity on the central nervous system, such as *Psychotria viridis*, popularly known as “ayahuasca” which means “soul wine”. *P. viridis* is used in religious ceremonies in association with *Banisteriopsis caapi*, a species from the Malpighiaceae family [30,31]. Their hallucinogenic effect is due to the synergy that occurs between the alkaloid *N*,*N*-dimethyltryptamine (DMT), present in the leaves of *P. viridis*, and β-carboline indole alkaloids (harmine, harmaline and tetrahydroharmine) present in the bark of *B. caapi* [32]. *Cephaelis* is another important genus, especially *C. ipecacuanha*, a plant traditionally used by the Brazilian population, an important source of emetine, an alkaloid with emetic, antihelminthic and expectorant effects [33,34]. In Brazil, species of *Palicourea* are considered responsible for about half of all cattle deaths brought about by natural poisoning [35]. Some selected isolated compounds from Rubiaceae species are shown in Table 1 and Figure 2.

## 4. Chemotaxonomic Considerations

Chemotaxonomic studies use chemical characteristics, particularly secondary metabolites from a group of organisms to determine their taxonomic classification [36]. This correlation between phytochemical compounds and morphological data becomes an important tool to determine plant classification, phylogeny and evolution [37,38,39].

The plant evolution process, from a morphological point of view, occured by the successive appearance of small weeds, larger herbs, shrubs and, finally, trees achieving the climax with primitive angiosperms. Then, the evolutionary polarity became inverted, woody plant being gradually replaced by herbaceous plants [40,41]. As explained by Gottlieb: “The most conspicuous evolutionary trend in the gross morphology of land plants concerns the successive appearance of small weeds, larger herbs, shrubs and, finally, trees. This trend had attained or even had passed its climax with the primitive angiosperms and within this division the evolutionary polarity became inverted, woody plants being gradually replaced by herbaceous plants.

**Table 1:** Some metabolites isolated from Rubiaceae.

Genera	Class	Substance	Structure *
*Cephaelis*	Alkaloid	Emetine	I
	Lactone	Chelidonic acid	II
	Alkaloid	Cephalin	III
	Alkaloid	Psycotrin	IV
*Cinchona*	Alkaloid	Quinine	V
	Triterpene	Cincholic acid	VI
	Triterpene	Quinovic acid	VII
	Alkaloid	Quinidine	VIII
	Alkaloid	Cinchonine	IX
	Alkaloid	Cinchonidine	X
*Coffea*	Methyl xantine	Caffeine	XI
	Diterpene	Cafestol	XII
	Anthraquinone	Galiosin	XIII
	Anthraquinone	Copareolatin	XIV
	Anthraquinone	Munjistin	XV
*Corynanthe*	Alkaloid	Yohimbine	XVI
*Galium*	Iridoide	Macedonine	XVII
*Genipa*	Monoterpene	Genipin	XVIII
*Hedyotis*	Anthraquinone	Alizarin	XIX
*Landerbergia*	Alkaloid	Quinidine	VIII
	Alkaloid	Cinchonine	IX
	Alkaloid	Cinchonidine	X
*Morinda*	Anthraquinone	Alizarin	XIX
*Mussaenda*	Triterpene	Arjunolic acid	XX
*Oldenlandia*	Anthraquinone	Alizarin	XIX
*Psychotria*	Alkaloid	Psycotrin	IV
	Alkaloid	Cephalin	III
*Relbunium*	Anthraquinone	Purpurin	XXI
*Remijia*	Alkaloid	Quinidine	VIII
	Alkaloid	Cinchonine	IX
	Alkaloid	Cinchonidine	X
*Rubia*	Anthraquinone	Purpurin	XXI
	Anthraquinone	Alizarin	XIX

* shown in Figure 2.

These successional phenomena are paralleled by micromolecular compositions. The ubiquitous flavonoids excepted, polyketides and terpenoids dominate the chemical compositions of bryophytes and pteridophytes. Shikimate-derived aromatics became numerically significant only in gymnosperms and attain predominance over other biosynthetic classes in primitive angiosperms. Concomitantly, here secondary metabolism reflects the trend from woody to herbaceous forms by inactivation of cinnamoyl/cinnamyl-derivatives through two phenomena: (i) *extension* of the shikimate pathway by reduction of cinnamyl alcohols to allylphenols and propenylphenols and (ii) gradual curtailment of the final steps of the shikimate pathway. The former alternative is most frequent in the primitive magnolialean block, where oxidative oligomerization of the precursors leads to neolignans. The first consequence of the latter alternative, the accumulation of phenylalanine and tyrosine, again very frequent in the magnolialean block, occurs also in the rosiflorean block. Oxidative elaboration of these amino acids leads to benzylisoquinolines. Further shortening of the shikimate pathway is restricted to the rosiflorean block. It leads to the accumulation of chorismic acid, the precursor of anthranilate- and of tryptophane-derived alkaloids, and of shikimic acid, the precursor of gallic acid- and ellagic acid-derived tannins. With gallic acid, the possibilities of diversifying the production of micromolecules through gradual curtailment of the shikimate pathway seem to be exhausted. In the most highly advanced, mostly sympetalous, angiosperms, shikimate-derived secondary metabolites play a relatively minor role. In these lineages, the full potential of acetate utilization leads to polyacetylenes, while mevalonate utilization leads to steroidal alkaloids, iridoids, alkaloids, sesquiterpene lactones, *etc.* In comparison with the polyketides and terpenoids of less advanced plant groups mentioned above, these compounds all show a high state of oxidation.” [40].

Regarding the distribution of the major secondary metabolites in Rubiaceae, indole alkaloids are indicated as the main chemical markers of this family [42,43,44,45,46]. Iridoids, anthraquinones, triterpene glycosides, flavonoids, lignoids, terpenes and phenols derivatives, were also reported [47]. Indole alkaloids occur just in families belonging to the Gentianales order (Loganiaceae, Rubiaceae, Apocynaceae and Naucleaceae), where one observes monoterpene indole alkaloids mainly [48]. The occurrence of indole alkaloids out of Gentianales order is quite rare and when found they are usually simple indole alkaloids.

A good correlation between the biosynthetic pathways and morphological aspects of the Ixoroideae, Cinchonoideae and Rubioideae subfamilies is obtained by evaluating chemical data, combined with the parameters cited by Robbrecht [8]. Each one of these subfamilies presents a different and typical profile of indole alkaloids, iridoids and anthraquinones which are considered as Rubiaceae chemotaxonomic markers [49]. Other studies based on chemotaxonomic data obtained by gas chromatography coupled to mass spectrometry show that the iridoid glycosides are present in several different species belonging to the Rubiaceae subfamilies [50,51,52]. Monoterpene indole alkaloids, especially which are derivatives of tryptamine and monoterpene (iridoid) secologanin are another predominant class in Rubiaceae. Quinoline alkaloids, which are products from the monoterpene indole and isoquinoline alkaloids rearrangement, yielding emetine-type alkaloids, are also characteristic of Rubiaceae, however, strychnine class alkaloids are not present in this family. Other alkaloid types are quite heterogeneous leading to a hard chemotaxonomic correlation [53].

Several studies have reported the use of chemical data to assist plant taxonomy [53]. Interest in this area increased due to the appearance of fast and accurate analytical techniques. However, there are still limitations on the application of chemical data in systematics. Even with a growing number of phytochemical studies, there are still many plants that remain without any chemical study.

## 5. Data Obtained Through the Bibliographic Survey

The present study sought to survey phytochemical studies of all species of Rubiaceae published in ScienceDirect and CAS SciFinder websites between 1990 and 2014. The data compiled in this review show the distribution of the studied species classified by their respective tribes and subfamilies as well as the isolated compounds and their chemical classes (Table 2).

Based on the obtained data, the main occurrence of iridoids, anthraquinones, triterpenes, indole alkaloids and alkaloids belonging to different chemical subclasses, was observed. The chemical profile, as expressed by the occurrence of major categories of secondary metabolites (alkaloids, anthraquinones and iridoids) showed to be quite different for each subfamily. Furthermore, the study of specific classes may contribute to chemotaxonomic correlations, since there are compounds with restricted distribution [54]. These same classes of substances served as a distribution pattern to create and modify plant classification systems as proposed by Dahlgren [54].

**Figure 3:**
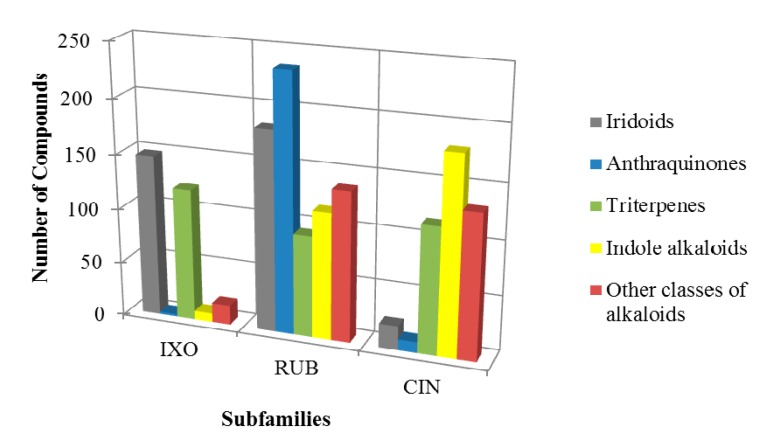
Chemical diversity and major secondary metabolites distribution among Rubiaceae subfamilies observed in this revision. IXO: Ixoroideae, CIN: Cinchonoideae, RUB: Rubioideae.

In Ixoroideae subfamily, the iridoids are found as chemotaxonomic markers, in Cinchonoideae the indole alkaloids predominate over other substances and in Rubioideae the anthraquinones are the major class of secondary metabolites (Figure 3). These global findings corroborate those found in the Brazilian Rubiaceae chemotaxonomic study by Bolzani [15].

Other studies also describe indole alkaloids as the class of substances of major occurrence in Cinchonoideae, especially in Guettardeae tribe [50,55]. Studies by Wijinsma and Verpoorte [56] and Bolzani *et al.* [15] describe the occurrence of standardized chemical markers: iridoids in Ixoroideae; indole alkaloids in Cinchonoideae and anthraquinones in Rubioideae. These data corroborate the one presented in this review.

Therefore, it was observed triterpenes widely distributed in all subfamilies, therefore a chemotaxonomic correlation cannot be established. The occurrence of a common pattern in secondary metabolism may suggest, strongly, taxons having a common ancestor. Thus, if there are morphological similarities, they can either be due to a common ancestry or convergent evolution [54]. Furthermore, the seco-iridoids are iridoids precursors and also participate in the biosynthesis of monoterpene indole alkaloids, so they may be involved in two distinct chemotaxonomic subdivisions [57,58]. Thus, different species may exhibit different chemical substance classes, but having the same precursor, which may indicates a phylogenetic relationship [59,60,61,62,63,64].

**Table 2:** Compounds isolated from Rubiaceae species, organized by subfamily and tribe.

Subfamily	Tribe	Species	Compound (s)	References
Cinchonoideae	CHI	*Chiococca alba*	*Triterpene glycosides*: chiococcasaponins I–V	[65]
			*Cetoalcohols*: 4-hydroxy-heptadecan-7-one; 5-hydroxy-octadecan-11-one *Phenylcoumarines*: 5,7,4′-trimethoxy-4-phenylcoumarine *Lignans*: exostemin; matairesinol; d-mannitol	[66]
			*Seco-iridoids*: albosides I–III	[67]
			*Nor*-*seco*-*pimarane*: merilactone	[68]
			*Triterpene*: 3-β-hydroxyolean-12,15-dien-28-oic acid	[69]
			*Triterpene glycosides*: *O*-α-d-apiofuranosyl (1→3)-[α-d-apiofuranosyl (1→4)]-α-l-rhamnopyranosyl (1→2)-α-l-arabinopyranosyl 3-*O*-β-d-glucopyranosyl-3-β-hydroxyolean-12,15-dien-28-oate; 28-*O*-α-d-apiofuranosyl (1→3)-α-l-rhamnopyranosyl (1→2)-α-l-arabinopyranosyl 3-*O*-β-d-glucopyranosyl-3-β-hydroxyolean-12,15-dien-28-oate	[70]
			*Ent-kaurane diterpenes*: 1-hydroxy-18-nor-kaur-4,16-dien-3-one; 15-hydroxy-kaur-16-en-3-one; kaur-16-en-19-ol; kaurenoic acid; merilactone; ribenone	[71]
			*Ent-kaurane: ent*-17-hydroxy-16α-kauran-3-one	[72]
		*Chiococca braquiata*	*Flavonoids*: 4′-methoxykaempferol-7-(acetyloxy)-3,5-*O*-α-l-rhamnoside; apigenin; 7-*O-*methoxyquercetrin; quercetrin *Triterpenes:* α-amirin; β-amirin; ursolic acid; oleanolic acid	[73]
		*Coutarea hexandra*	*Coumarins:* 5-*O*-β-d-glucopyranosyl-4-(4-hydroxyphenyl)-7-methoxy-2*H*-chromen-2-one; 5-*O*-β-d-galactopyranosyl-4-(4-hydroxyphenyl)-7-methoxy-2*H*-chromen-2-one *Cucurbitacins*: 23,24-dihydrocucurbitacin F; 23,24-dihydro-25-acetylcucurbitacin F; 2-*O*-β-d-glucopyranosyl-23,24-dihydrocucurbitacin F	[74]
		*Exostema acuminatum*	*Nor*-*diterpenes:* *ent*-16,17-diidroxicauran-19-nor-4-en-3-one; *ent*-16,17-dihydroxy-kauran-19-nor-4-en-3-one *Phenylcoumarins*: 5,7,4′-trimethoxy-4-phenylcoumarin; 7,4′-dimethoxy-5-hydroxy-4-phenylcoumarin; 5,7,4′-trimethoxy-3′-hydroxy-4- phenylcoumarin; 5,7,4′-trimethoxy-8-hydroxy-4-phenylcoumarin (exostemin I); 5,7,4′-trimethoxy-8,3′-dihydroxy-4′-phenylcoumarin;	[75]
			7,4′-dimethoxy-5,3′-hydroxy-4′-phenylcoumarin	[75]
		*Exostema caribaeum*	*Phenylcoumarin*: 5-*O*-β-d-galactopyranosyl-7-methoxy-3′, 4′-dihydroxy-4-phenylcoumarin	[76]
		*Hintonia latiflora*	*Phenylcoumarin*: 5-*O*-(6′′acetyl-β-d-glucopyranosyl)-7,3′,4′-trihydroxy-4-phenylcoumarin *Phenylstyrene*: 6-*O*-β-d-glucopyranosyl-2,3′,4β-trihydroxy-4-methoxy-β-phenylstyrene	[77]
		*Hintonia standleyana*	*Phenylcoumarin*: 3-*O*-β-d-glucopyranosyl-23,24-dihydrocucurbitacin F; 5-*O*-[β-d-apiofuranosyl-(1→6)-β-d-glucopyranosyl]-7-methoxy-3′,4′-dihydroxy-4-phenyl-coumarin; desoxycordifolinic acid	[78]
	CIN	*Cinchona ledgeriana*	*Quinolinic alkaloids:* quinine; quinidine; cinchonidine and cinchonine	[79,80]
		*Cinchona robusta*	*Anthraquinones*: robustaquinones A–H; 1,3,8-trihydroxy-2-methoxyanthraquinone; copareolatin 6-methyl ether	[81]
		*Ladenbergia oblongifolia*	*Alkaloids*: epicinchonicinol; cinchonidicinol; mixture of dihydrocinchonicinol and dihydrocinchonidicinol	[82]
		*Remijia peruviana*	*Quinolinic alkaloids*: quinine; cuprein; cinchonine; acetylcupreine; *N*-ethylquinine	[83]
			*Alkaloids:* remijinine; epiremijinine; 5-acetylapocinchonamine; *N*-acetyldeoxy-cinchonicinol; *N*-acetylcinchonicinol	[84]
		*Sickingia tinctoria*	*Indole alkaloids:* sickingin; 5-carboxystrictosidine; ophiorines A–B; lyalosidic acid	[85]
		*Sickingia williamsii*	*Indole alkaloids*: sickingin; 5α-carboxystrictosidine; ophiorines A–B; lyalosidic acid	[85]
	GUE	*Antirhea acutata*	*Triterpene-methyl ester*: *nor*-*seco*-cycloartane	[86]
		*Antirhea lucida*	*Indole alkaloids*: *N*,*N*-methyl-3′-indolylmethyl-5-methoxytryptamine; *N*,*N*-dimethyltryptamine; 6-methoxy-2-methyl-1,2,3,4-tetrahydro-13-carboline	[87]
		*Antirhea portoricensis*	*Indole alkaloids*: 20-epiantirhine; isoantirhine; antirhine; yohimbol; epi-yohimbol; 19(*S*)-hydroxydihydrocorinanteol	[88]
		*Chomelia obtusa*	*Triterpenes*: 3-*O*-β-d-quinovopyranosyl-28-*O*-β-d-glycopyranosyl quinovic acid; 3-*O*-β-d-quinovopyranosyl-28-*O*-β-d-glycopyranosyl cincholic acid; ursolic acid; oleanolic acid *Flavonoids*: (3-*O*-β-d-glycopyranosyl quercetin; 3-*O*-[α-l-rhamnopyranosyl-(1→6)-β-d-galactopyranoside] quercetin; 3,5-*O*-dicaffeoyl quinic acid; 4,5-*O*-dicaffeoyl quinic acid	[89]
		*Guettarda grazielae*	*Triterpenes*: α-amyrin acetate; cycloartenone; 3β,19α,23-trihydroxyurs-12-ene; 3-β-*O*-β-d-glucopyranosylquinovic acid; 3β,6β,19α,23-tetrahydroxyurs-12-en-28-oic; acid ursolic acid	[90]
			*Iridoid*: guettardodiol *Seco-iridoid:* sarracenin; 7α-morroniside; 7β-morroniside	[91]
		*Guettarda noumeana*	*Quinolinic alkaloids*: cupreine; dihydrocupreine; *N*-methyldihydroquinicinol; *N*-methylquinicinol	[92]
		*Guettarda pohliana*	*Triterpenes*: ursolic acid; oleanolic acid; pomolic acid; rotundic acid; 3β,6β,19α,23-tetra-hydroxyurs-12-en-28-oic acid; clethric acid *Monoterpene*: 5-*O*-caffeoylquinic acid; loliolide *Seco-iridoid*: secoxiloganin	[93]
			*Triterpenes glycosides:* 28-*O*-β-d-glycopyranosyl-3-*O*-β-d-quinovopyranosyl quinovic acid; 28-*O*-β-d-glycopyranosyl-3-*O*-β-d-glycopyranosyl quinovic acid; 3-*O*-β-d-glycopyranosyl quinovic acid; 28-*O*-β-d-glycopyranosyl-3-*O*-β-d-glycopyranosyl cincholic acid; quinovic acid; daucosterol *Phenolic compound:* 4,5-*O*-dicaffeoylquinic acid	[94]
		*Guettarda speciosa*	*Phenolic compounds:* 1-*O*-α-d-glucuronide 3-*O*-benzoyl ester; guettardionoside *Indole alkaloid*: cadambine *Iridoid glycoside:* sweroside; morroniside *Steroids*: ecdysone; icariside D_1_ *Triterpene*: quinovic glycoside C	[95]
		*Machaonia brasiliensis*	*Steroids:* 3β-*O*-β-glucopyranosyl stigmasterol; 3β-*O*-β-glucopyranosyl sitosterol *Seco-iridoid*: secologanoside *Flavonoid*: 7-*O*-β-glucopyranosyl quercetagetin *Clorogenic acids*: 4,5-*O*-dicaffeoylquinic acid; 5-*O*-caffeoylquinic acid.	[96]
		*Neolamarckia cadamba*	*Indole alkaloids*: neolamarckines A–B	[97]
		*Neolaugeria resinosa*	*Oxindole alkaloids*: neolaugerine; isoneolaugerine; 15-hydroxyneolaugerine	[98]
		*Timonius timon*	*Triterpenes:* 3β,6β,23-trihydroxy-olean-12-en-28-oic acid; 3β,6β,19α,23-tetrahydroxy-olean-12-en-28α-oic acid	[99]	
	HAM/HIL	*Chione venosa* var. *venosa*	*Acetophenone derivatives*: ortho-hydroxy-acetophenone-azine; acetophenone-2-*O*-β-d-glucopyranoside; acetophenone-2-*O*-[β-d-apiofuranosyl-(1→6′)-*O*-β-d-glucopyranosyl] *Iridoid glycosides*: 4α-morroniside; sweroside; diderroside *Triterpene*: daucosterol	[100]
	HAM	*Deppea blumenaviensis*	*β-carboline alkaloids*: deppeaninol	[101]
		*Hamelia magniflora*	*Indole alkaloids*: magniflorine; ajmalicine	[102]
		*Hamelia patens*	*Indole alkaloids*: (−)-hamelin; tetrahydroalstonin; aricine; pteropodine; isopteropodine; uncarine F; speciophylline; palmirine; mitraphylline; rumberine	[103]
	HYM	*Hymenodictyon excelsum*	*Triterpenes:* 3β-hydroxy-11-oxours-12-en-28-oic acid; 3β-hydroxy-27-*p*-(*Z*)-coumaroyloxyolean-12-en-28-oic acid; 3-oxo-11α,12α-epoxyurs-13β,28-olide; 3β-hydroxy-11α,12α-epoxyurs-13β,28-olide; 3β-hydroxyurs-11-en-13(28)-lactone; oleanolic acid; uncarinic acid E (3β-hydroxy-27-(*E*)-*p*-coumaroyloxyolean-12-en-28-oic acid; ursolic acid; ursonic acid; 3β-(formyloxy)-urs-12-en-28-oic acid	[104]
		*Hymenodictyon floribundum*	*Glycosides:* scopolin; himexelsin or xeroboside; scopoletin	[105]
			*Iridoids*: floribundane A–B	[106]
	ISE	*Isertia haenkeana*	*Indole alkaloids*: dihydroquinamine; epidihydroquinamine; apodihydrocinchonamine; 3-carbomethoxy-5-(l′-hydroxyethyl) pyridine	[107]
		*Isertia pittieri*	*Triterpene glycosides*: pyrocincholic acid 3β-*O*-α-d-quinovopyranosyl-28-[β-d-glucopyranosyl(1→6)-β-d-glucopyranosyl] ester; pyrocincholic acid 3β-*O*-β-d-quinovopyranosyl(1→6)-α-d-glucopyranosyl-28-[-β-d-glucopyranosyl(1→2)-β-d-glucopyranosyl] ester; quinovic acid 3α-*O*-*R*-l-rhamnopyranosyl(28→1)-β-d-glucopyranosyl ester; quinovic acid 3β-*O*-β-d-glucopyranosyl(1→4)-*R*-l-rhamnopyranosyl-(28→1)-β-d-glucopyranosyl ester	[108]
	NAU	*Adina cordifolia*	*Coumarins*: umbelliferone; skimmin; 7-methoxycoumarin and 7-hydroxy-8-acetyl coumarin	[109]
		*Adina racemosa*	*Flavonoid glycosides:* quercetin 3-*O*-*R*-l-rhamnopyranosyl(16)-(3-*O*-*trans*-*p*-coumaroyl)-α-d-galactopyranoside; quercetin 3-*O*-*R*-l-rhamnopyranosyl(1→6)-[(4-*O*-*trans*-*p*-coumaroyl)-*R*-l-rhamnopyranosyl(1→2)]-(4-*O*-*trans*-*p*-coumaroyl)-α-d-galactopyranoside; kaempferol 3-*O*-*R*-l-rhamnopyranosyl(1→6)-[(4-*O*-*trans*-*p*-coumaroyl)-*R*-l-rhamno-pyranosyl(1→2)]-(4-*O*-*trans*-*p*-coumaroyl)-β-d-galactopyranoside; quercetin 3-*O*-*R*-l-rhamnopyranosyl(1→6)-[(4-*O*-*trans*-*p*-coumaroyl)-*R*-l-rhamnopyranosyl(1→2)]-(3-*O*-*trans*-*p*-coumaroyl)-β-d-galactopyranoside; quercetin 3-*O*-*R*-l-rhamnopyranosyl(1→6)-[(4-*O*-*trans*-caffeoyl)-*R*-l-hamnopyranosyl-(1→2)]-(3-*O*-*trans*-*p*-coumaroyl)-β-d-galactopyranoside	[110]
			*Secoiridoid glucosides:* adinosides A–E; grandifloroside 11-methyl ester	[111]
		*Adina rubella*	*Triterpenes glycosides:* quinovic acid 3-*O*-β-d-glucopyranosyl (l→4)-β-d-fucopyranoside; quinovic acid 3-*O*-β-d-glucopyranosyl (1→4)-β-d-fucopyranoside (28→1)-β-d-glucopyranosyl ester; quinovic acid 3-*O*-β-d-glucopyranosyl (1→4)-α-l-rhamnopyranosyl-(28→1)-β-d-glucopyranosyl ester; quinovic acid 3-*O*-β-d-glucopyranosyl (1→2)-β-d-glucopyranosyl-(28→1)-β-d-glucopyranosyl ester	[112]
			*27-Nor-triterpene glycosides*: rubellosides C–D	[113]
		*Adina polycephala*	*Iridoids*: genipin-1-*O*-α-l-rhamnopyranosyl (1→6)-α-d-glucopyranoside	[114]
		*Cephalanthus glabratus*	*Oxindole alkaloids*: tetrahydroalstonine; mitraphylline; uncarine E	[115]
		Cephalanthus occidentalis	*Triterpenes glycosides:* 3-*O*-α-glucopyranosylcincholic acid; cincholic acid 28-*O*-α-glucopyranosyl ester; 3-*O*-β-glucopyranosyl-(1→4)-β-fucopyranosylcincholic acid; 3-*O*-β-glucopyranosyl-(1→4)-β-fucopyranosylcincholic acid 28-*O*-β-glucopyranosyl ester; 3-*O*-β-glucopyranosylcincholic acid 28-*O*-α-arabinopyranosyl-(1→2)-β-glucopyranosyl ester; 3-*O*-β-glucopyranosylquinovic acid 28-*O*-α-arabinopyranosyl-(1→2)-β-glucopyranosyl ester	[116]
		*Corynanthe pachyceras*	*Indole alkaloids*: corynanthine; α-yohimbine; dihydrocorynanthine; corynantheine; corynantheidine	[117]
		*Mitragyna diversifolia*	*Monoterpe indole alkaloids*: mitradiversifoline; specionoxeine-*N*(4)-oxide; 7-hydroxyisopaynantheine; 3-dehydropaynantheine; 3-isopaynantheine-*N*(4)-oxide	[118]
		*Mitragyna inermis*	27-*Nor*-*glycosides triterpene:* inermisides I–II *Triterpenes*: quinovic acid; 3-*O*-[β-d-glucopyranosyl-(1→4)-α-l-rhamnopyranosyl]; β-d-glucopyranosyl-[3-*O*-(β-d-glucopyranosyl)]-quinovic acid; 3-*O*-(β-d-6-deoxy-glucopyranosyl) quinovic acid	[119]
			*Indole alkaloids:* naucleactonin D; nauclefilline; angustoline; angustine; naucleficine; nauclefidine *Triterpenes*: barbinervic acid; quinovic acid; 3-*O*-*α*-l-rhamnopyranoside acid; betulinic acid; oleanolic acid; ursolic acid; strictosamide	[120]
			*Oxindole alkaloids*: mitraphylline; isomitraphylline; speciophylline; pteropodine	[121]
		*Mitragyna parvifolia*	*Oxindole alkaloids:* 16,17-dihydro-17β-hydroxyisomitraphylline; 16,17-dihydro-17β-hydroxymitraphylline; 2-isomitraphylline; mitraphylline	[122]
		*Mitragyna rotundifolia*	*Triterpene glycosides*: quinovic acid 3-*O*-β-d-6-deoxy-glucopyranoside 28-*O*-β-d-glucopyranosyl ester; quinovic acid 27-*O*-α-l-rhamnopyranosyl ester; 3-*O*-α-l-rhamnopyranoside; quinovic acid 27-*O*-β-d-glucopyranosyl ester; quinovic acid 3-*O*-6-deoxy- glucopyranoside; quinovic acid 27-*O*-β-d-glucopyranosyl ester; cincholic acid 3-*O*-β-d-6-deoxy-glucopyranoside; cincholic acid 28-*O*-β-d-glucopyranosyl ester	[123]
		*Mitragyna speciosa*	*Indole alkaloids*: mitragynine; speciogynine; speciociliatine; 7-hydroxy-mitragynine; paynantheine	[124]
		*Nauclea cadamba*	*Gluco-indole alkaloids*: 3β-dihydroisocadambine; cadambine; 3α-dihydrocadambine; 16-carbomethoxynaufoline; nauclechine; 5,11,12,5α-tetrahydroindolo[3,2-*g*]-pyridino-[4,3-*b*]indolizine	[125]
		*Nauclea diderrichii*	*Triterpene glycosides:* quinovic acid 3-*O*-α-l-rhamnopyranosyl (28→1)-β-d-gluco-pyranosyl ester; quinovic acid 3-*O*-β-d-glucopyranosyl (1→2)-d-glucopyranoside; quinovic acid 3-*O*-β-l-fucopyranosyl (28→1)-β-d-glucopyranosyl ester	[126]
			*Indole alkaloids*: 3α-5α-tetrahydrodeoxycordifoline; cadambine acid	[127]
		*Nauclea latifolia*	*Indole alkaloids:* latifoliamides A–E; angustoline	[128]
		*Nauclea officinalis*	*Indole alkaloids:* naucleficines A–E; naucleidinal; angustoline	[129]
			*Indole alkaloids*: naucline; angustine; angustidine; nauclefine; naucletine	[130]
			*Triterpenes*: 3β,19α,23,24-tetrahydroxyurs-12-en-28-oic acid; 2β,3β,19α,24-tetrahydroxyurs-12-en-28-oic acid; 3-oxo-urs-12-ene-27; 28-dioic acid; quinovic acid 3-β-rhamnopyranoside	[131]
		*Nauclea orientalis*	*Tetrahydro-β-carboline monoterpene alkaloid glucosides*: naucleaorine; epimethoxynaucleaorine; strictosidine lactam *Triterpenes:* oleanolic acid; 3,4,5-trimethoxyphenol; 3-hydroxyurs-12-en-28-oic acid methyl ester; 3α,23-dihydroxyurs-12-en-28-oic acid; 3α,19α,23-trihydroxyurs-12-en-28-oic acid methyl ester	[132]
			*Indole alkaloids*: nauclealines A–B; naucleosides A–B; strictosamide; vincosamide; pumiloside	[133]
			*Indole alkaloids:* naucleaorals A–B	[134]
		*Nauclea pobeguinii*	*Indole alkaloids:* naucleidinal; magniflorine; naucleofficine D; diastereoisomers of 3,14-dihydroangustoline; strictosidine; desoxycordifoline; 3α,5α-tetrahydrodeoxycordifoline lactam *Phenolic compound*: kelampayoside A	[135]
			*Indole alkaloid:* nauclequinine; nauclefoline; nauclefidine	[136]
		*Neonauclea purpurea*	*Quinolinic alkaloid*: 2,6-dimethoxy-1,4-benzoquinone	[137]
			*Indole alkaloids*: cadambine; α-dihydrocadambine	
		*Neonauclea sessilifolia* **	*Triterpene glycosides:* 3-*O*-β-d-glucopyranosyl quinovic acid; 3-*O*-β-d-glucopyranosyl-(1→2)-β-d-quinovopyranosyl quinovic acid; 3-*O*-β-d-quinovopyranosyl pyrocincholic acid 28-*O*-β-d-glucopyranosyl-(1→6)-β-d-glucopyranosyl ester; 3-*O*-α-l-rhamnopyranosyl-(1→4)-β-d quinovopyranosyl pyrocincholic acid 28-*O*-β-d-glucopyranosyl-(1→6)-β-d-glucopyranosyl ester	[138]
			*Triterpene:* ursolic acid	[139]
			*Chromone-secoiridoid glycosides*: sessilifoside; 7′′-*O*-β-d glucopyranosylsessilifoside *Indole alkaloid glycosides*: neonaucleosides A–C *Glycosides:* 5-hydroxy-2-methylchromone-7-*O*-β-d-apiofuranosyl-(1→6)-β-d-glucopyranoside; sweroside; loganin; grandifloroside; quinovic acid 3β-*O*-β-d-quinovopyranoside-28-*O*-β-d-glucopyranoside	[140]
		*Ochreinauclea maingayii*	*Indole alkaloids*: neonaucline; cadamine; naucledine	[141]
		*Pausinystalia johimbe*	*Monoterpene indole alkaloid:* yohimbine	[142]
		*Uncaria attenuata*	*Oxindole alkaloids:* corynoxine; corynoxine B; isocorynoxeine; *epi-allo*-corynantheine; dihydrocorynantheine pseudoindoxyl *Indole* *alkaloids*: 19-*epi*-3-*iso*-ajmalicine *Triterpene*: ursolic acid	[19]
		*Uncaria borneensis*	*Alkaloids*: isorhynchophylline; rhynchophylline; isocorynoxeine; corynoxeine; *Indole alkaloids*: *allo*-yohimbine; *pseudo*-yohimbine; 3-*epi*-β-yohimbine	[143]
		*Uncaria callophylla*	*Indole alkaloids:* dihydro-corynantheine; gambirine; isogambirine; gambireine; rotundifoline; callophylline; callophyllines A–B; yohimbine; pseudoyohimbine; β-yohimbine; α-yohimbine	[144]
			*Indole alkaloids:* callophyllines A–B; 3-*epi*-β-yohimbine; gambirine	[144]
		*Uncaria cordata* var. *cordata* and *Uncaria cordata* var. *ferruginea*	*Indole alkaloids:* dihydrocorynantheine	[143]
		*Uncaria elliptica*	*Pentacyclic oxindole alkaloids*: formosanine; isomitraphylline; mitraphylline *Indole alkaloids*: ajmalicine	[145]
			*Triterpenes:* 3β,6β,19α-trihydroxy-23-oxo-urs-12-en-28-oic acid; 3β,6β,19α,23-trihydroxy-23-oxo-urs-en-28-oic acid; 3,6-dioxo-19α-hydroxy-urs-12-ene-28-oic acid; 3β,6β-diacetoxi-19-hydroxy-urs-12-ene-28-oic acid; quinovic acid 3β-*O*-β-d-quinopyranosyl-(28→1)-β-d-glucopyranosyl ester	[145]
		*Uncaria gambir*	*Proanthocyanidins*: gambiriins A1–A2 ; gambiriins B1–B2; (+)-catechin; (+)-epicatechin; procyanidin B1; procyanidin B3; gambiriin	[146]
		*Uncaria glabrata*	*Monoterpene indole alkaloids*: 14α-hydroxyrauniticine; rauniticine; uncarine C–E; glabratine; deoxycordifoline	[147]
		*Uncaria guianensis*	*Indole alkaloid*: 3-isoajmalicine *Oxindole alkaloids*: isomitraphylline; mitraphylline; isomitraphylinic acid	[38]
			*Indole alkaloid*: ajmalicine *Oxindole alkaloids*: formosanine or uncarine B; isomitraphylline; mitraphylline	[148]
			*Triterpenes*: quinovic acid 3β-*O*-β-d-quinovopyranoside; quinovic acid 3β-*O*-β-d-fucopyranosyl-(27→1)-β-d-quinovopyranosyl ester; quinovic acid 3β-*O*-[β-d-glucopyranosyl-(1→3)-β-d-fucopyranosyl]-(27→1)-β-d-glucopyranosyl ester; quinovic acid 38-*O*-β-d-fucopyranoside	[149]
		*Uncaria hirsuta*	*Bis(monoterpenoid) indole alkaloid glucosides*: hirsutaside D; bahienoside A–B; neonaucleoside B	[150]
			*Phenolic compound:* chlorogenic acid *Alkaloid*: uncarine B *Flavonoids*: quercitrin; rutin; hiperin; neohesperidin	[151]
		*Uncaria lanosa* var. *glabrata* and *Uncaria lanosa* var. *ferrea*	*Pentacyclic oxindole alkaloids:* isopteropodine; pteropodine	[143]
		*Uncaria longiflora* var. *longiflora*	*Alkaloids:* isorhynchophylline; rhynchophylline; iso-corynoxeine; corynoxeine	[143]
		*Uncaria longiflora* var. *pteropoda*	*Pentacyclic oxindole alkaloids:* pteropodine; isopteropodine	[143]
			*Pentacyclic oxindole alkaloids:* pteropodine; isopteropodine	[152]
		*Uncaria macrophylla*	*Oxindole alkaloids:* rhynchophylline; isorhynchophylline; corynoxine; corynoxine B	[153]
		*Uncaria rhynchophylla* **	*Indole alkaloids:* tetrahydroalstonine; tetrahydroalstonine-*N*-oxide; akuamigine; (4*R*)-akuamigina-*N*-oxide; (4*S*)-akuamigine-*N*-oxide; corynantheine; dihydrocorynantheine; dihydrocorynantheine-*N*-oxide; hirsuteine; geissoschizine methyl ether; hirsutine *N*-oxide; akuamigine pseudoindoxyl; rauniticine pseudoindoxyl; 3-isorauninticine pseudoindoxyl; dihydrocorynantheine pseudoindoxyl; vallesiachotamine; vincoside lactam; strictosamide; rhynchophyne; 2′-*O*-β-d-glucopyranosyl-11-hydroxyvincoside lactam; angustine; angustoline; angustidine	[154]
			*Sesquiterpene indole alkaloids:* (5*S*)-5-carboxystrictosidine; 3,4-dehydro-(5*S*)-5-carboxystrictosidine *Indole alkaloids:* cadambine; 3α-dihydrocadambine; 3β-isodihydrocadambine *Pentacyclic oxindole alkaloids*: isorhynchophylline; rhynchophylline; corynoxeine; isocorynoxeine; corynoxeine; rhynchophylline *N*-oxide; isorhynchophylline *N*-oxide; macrophylline A; 18-19-dehydrocorynoxinic acid; 22-*O*-demethyl-22-*O*-β-d-glucopyranosyl isocorynoxeine	[154]
			*Oxindole alkaloids*: rhynchophylline; corynoxeine; corynanteine; hirsutine	[155]
			*Oxindole alkaloids*: isocorynoxeine; isorhynchophylline; orynoxeine; rhynchophylline *Indole alkaloids*: corynanteine; dihydrocorynanteine	[156]
			*Pentacyclic oxindole alkaloids*: 22*-O*-demethyl-22-*O*-β-glucopyranosyl isorhynchophylline; 22*-O*-demethyl-22-*O*-β-glucopyranosyl rhynchophylline; 22*-O*-demethyl-22-*O*-β-glucopyranosyl isocorynoxeine; isorhynchophylline acid; 9-hydroxy isocorynoxeine; 18,19-dehydrocorynoxinic acid; 18,19 dehydrocorynoxinic acid B; rhynchophyllic acid; 9-hydroxycorynoxeine; isocorynoxeine *N*-oxide; rhynchophylline acid *N*-oxide; corynoxeine *N*-oxide; isocorynoxeine; rhynchophylline; isorhynchophylline *N*-oxide; isorhynchophylline; corynoxeine *Indole alkaloid:* vincoside lactam *Phenolic compounds:* chlorogenic acid; neochlorogenic; cryptochlorogenic; quinic acid; *cis*-5-caffeoylquinic acid; procyanidin b1; procyanidin b2; catechin; *epi*-catechin; rutin	[157]
		*Uncaria salaccensis*	*Oxindole alkaloids:* 3-oxo-7-hydroxy-3,7-secorhynchophylline	[158]
		*Uncaria sinensis*	*Alkaloids*: isohynchophyllic acid; pteropodic acid; 3α-dihydrocadambine; 3β-isodihydrocadambine	[159]
			*Proanthocyanidin:* procyanidin B-1	[160]
		*Uncaria tomentosa* **	*Pentacyclic alkaloids*: isomitraphylline; mitraphylline; uncarine F; speciophylline; isopterophylline; pterophylline; isocorynoxeine *Tetratacyclic alkaloids*: corynoxeine; isorincophylline; rincophylline	[161]
			*Alkaloids:* cinchonain Ia; cinchonain Ib	[162]
			*Oxindole alkaloids:* uncarines C–E; mitraphylline; isomitraphylline *Iridoid glycosides:* 7-deoxyloganic acid	[163]
			*Triterpenes glycosides:* 3-oxo-6β-19α-dihydroxyurs-12-en-28-oic acid; 3β,6β,19α,23-tetrahydroxyurs-12-en-28-oic acid; 3β-methoxy-16α-hydroxyurs-12,19(29)-dien-27,28-dioic acid; 3β-hydroxyurs-12-en-27,28-dioic acid	[164]
			*Oxindole alkaloids*: pteropodine; isopteropodine; speciophylline; uncarine F; mitraphylline; isomitraphylline; rincophylline; isorincophylline	[165]
			*Oxindole alkaloids*: mitraphylline	[166,167]
			*Indole alkaloid:* 3-isoajmalicine	[168]
			*Alkaloids:* cinchonain Ia; cinchonain Ib	[162]
			*Iridoids:* tomentosides A–B *Phenolic compound*: (−)-*epi*-cathequin	[169]
			*Triterpenes:* oleanolic acid; 3β,6β,19α-trihydroxyurs-12-en-28-oic acid	[170]
			*Triterpenes:* 3β,6β,19α-trihydroxyurs-12-en-23-al-28-oic acid; 3β,19α-dihydroxy-6-oxo-urs-12-en-23-al-28-oic acid; 3β,19α-dihydroxy-6-oxo-urs-12-en-23-ol-28-oic acid	[171]
			*Triterpene:* 23-*nor*-24-esomethylene-3β,6β-19α-trihydroxyurs-12-en-28 oic acid; 3β,6β,19α-trihydroxyurs-12-en-28-oic acid; 3-oxo-6β,19α-dihydroxyurs-12-en-28 oic acid; oleanic acid	[169]
		*Uncaria villosa*	*Indole alkaloids*: villocarines A–D	[172]
Ixorideae	ALB	*Alberta magna*	*Iridoids*: (+)-5-acetaldehyde-l-formyl-2-methylcyclopentan; 5-acetaldehyde-1-formyl-2- methylcyclopent-1-ene; 1,4α,5,6,7α-hexahydro-1-hydroxy-7-methylcyclopenta-pyran-4-carboxaldeyde; 4,4α,5,7α-tetrahydro-1-hydroxy-4-(hydroxymethylene)-7-methylcyclopentane-pyran-3-(1*H*)-one; 5-deoxystansioside; 6,10-bisdeoxyaucubin; boschnaloside	[173]
	COF	*Coffea* sp	*Alkaloid*: caffeine	[174]
		*Coffea bengalensis*	*Alkaloid*: caffeine *Diterpene*: 16-epicafestol	[175]
		*Nematostylis anthophylla*	*Triterpene glycosides:* randianin; 2′′-*O*-acetylrandianin; 6′′-*O*-acetylrandianin	[176]
		*Tricalysia dubia*	*Diterpenes*: tricalysiol A–B; tricalysiolide B; tricalysioside G tricalysioside L	[177]
			*Ent-kaurane glycosides:* tricalysiosides A–G	[178]
		*Tricalysia okelensis*	*Ent-kaurane glycosides:* *ent*-kauran-3α,16α,17-triol-19-al 3-*O*-[5-*O*-vanilloyl-β-d-apiopyranosyl(1→6)]-β-d-glucopyranoside; *ent*-kauran-3α,16α,17-triol-19-al; 3-*O*-[5-*O*-E-sinapoyl-β-d-apiopyranosyl(1→6)]-β-d-glucopyranoside	[179]
	CON	*Calycophyllum spruceanum*	*Seco*-*iridoids*: 7-methoxydiderroside,6′-*O*-acetyldiderroside; 8-*O*-tigloyldiderroside; loganetin; loganin; secoxyloganin; kingiside; diderroside	[180]
		*Chimarrhis turbinata*	*Indole monoterpene alkaloids:* strictosidine; strictosidine acid; 5α-arboxystrictosidine; isovallesiachotamine; vallesiachotamine; turbinatine; 3,4-dehydro-strictosidine; turbinatine *β-Carboline alkaloids*: cordifoline; deoxycordifoline; harman-3-carboxylic acid	[181]
		*Crossopteryx febrifuga*	*Triterpene glycosides:* 3β-(α-l-rhamnopyranosyloxi)-28-*O*-(β-d-glucopyranosyl)urs-12,20(30)-diene-27,28-dioic acid	[182]
		*Emmenopterys henryi*	*Triterpenes:* 3β,19α,23-trihydroxyurs-12-en-24-al-28-oic acid; 3β,19α,24-trihydroxy-23-norurs-12-en-28-oic acid; 3β,12β-dihydroxy-5α-pregnane-14,16-dien-20-one; and 12β-hydroxy-5α-pregnane-14,16-dien-3,20-dione; 3β,19α,23,24-tetrahydroxyurs-12-en-28-oic acid; pomolic acid; 3β,6β,19α,23-tetrahydroxyurs-12-en-28-oic acid; 3β,6β,23-trihydroxyolean-12-en-28-oic acid; 3β,6β,19α,23-tetrahydroxyolean-12-en-28-oic acid; 3β,23,24-trihydroxyolean-12-en-28-oic acid; 3β,12β-dihydroxy-5α-pregnane-16-en-20-one; 12β-dihydroxy-5α-pregnane-16-en-3,20-dione	[183]
		*Pogonopus speciosus*	*Alkaloids:* 1′,2′,3′,4′-tetradehydrotubulosine; tubulosine; psychotrine	[184]
		*Pogonopus tubulosus*	*Alkaloid:* tubulosine	[185]
			*Alkaloids:* tubulosine; psychotrine; cephaeline	[186]
		*Simira glaziovii*	*Alkaloids*: aribin; ophiorine B; lyaloside *Monoterpenes:* methyl 3,4-dimethoxycinamate	[187]
		*Simira eliezeriana*	*Diterpenes:* simirane A [(5*R*,6*R*,8*R*,9*R*,10*S*,11*S*,13*S*)-6 β,11β -dihydroxy-2,4(18),15-erythroxylatrien-1-one]; simirane B [(5*S*,8*R*,9*R*,10*S*,11*S*,13*S*)-11-hydroxy-2,4(18),15-erythroxylatrien-1-one]	[188]
	GAR	*Alibertia edulis*	*Iridoids:* 6β-hydroxy-7-epigardoside methyl ester	[189]
		*Alibertia macrophylla*	*Diterpene: ent*-kaurane-2β,3α,16α-triol *Triterpenes*: lupenone; germanicone; α-amirenone; β-amirenone; lupeol; oleanolic acid; ursolic acid G*lucosidic iridoids*: 6α-hydroxygeniposide; 6β-hydroxygeniposide; gardenoside; shanziside methylester *Phenolic acids*: protocatechuic; vanilic; caffeic	[190]
		*Alibertia myrciifolia*	*Coumarin*: scopoletin	[64]
			*Flavonoid*: corymbosin	[191]
			*Iridoid*: 10-*O*-vanilloylgeniposidic acid	[192]
			*Triterpenes*: pomolic acid methyl ester; ursolic acid methyl ester; oleanolic acid methyl ester	[193]
		*Alibertia sessilis*	*Phenolic compounds*: 3,4,5-trimethoxyphenyl-1-*O*-β-d-(5-*O-*syringoyl)-apiofuranosyl-(1→6)-β-d-glucopyranoside *Iridoids*: geniposidic acid; geniposide; 6α-hydroxygeniposide; 6β-hydroxygeniposide *Lignans glycosides*: (+)-lyoniresinol-3α-*O*-β-d-glucopyranoside; (−)-lyoniresinol-3α-*O*-β-d-glucopyranoside	[64]
			*Flavonoids*: quercetin-3-*O*-β-d-(2′′-*O*-*trans*-*p*-coumaroyl)-rutinoside; kaempherol-3-*O*-β-d-(2′′-*O*-*trans*-*p*-coumaroyl)-rutinoside *Triterpenes*: oleanolic acid; ursolic acid; *epi*-betulinic acid *Iridoids:* gardenoside; deacetylasperuloside; 10-dehydrogardenoside; β-gardiol; α-gardiol	[46]
		*Burchellia bubalina*	*Iridoids*: β-gardiol; α-gardiol; garjasmine	[60]
		*Canthium gilfillanii*	*Iridoid*: geniposidic acid	[61]
		*Catunaregam nilotica*	*Triterpene glycosides:* 28-*O*-β-d-glucopyranosyl-3-*O*(*O*-α-l-rhamnopyranosyl-(1→3)-*O*-β-d-glucopyranosyl]-(1→3)]-β-d-glucopyranosyl) oleanolate; 3-*O*-[2′,3′-di-*O*-(β-d-glucopyranosyl)-β-d-glucopyranosyl] oleanolic acid; 3-*O*-(*O*-α-l-rhamnopyranosyl-(1→3)-*O*-[*O*-β-d-glucopyranosyl-(1→3)]-β-d-glucopyranosyl) oleanolic acid; 3-*O*-[*O*-β-d-glucopyranosyl-(1→3)-β-d-glucopyranosyl] oleanolic acid	[194]
		*Catunaregam spinosa*	*Triterpene glycosides:* catunarosides A–D; swartziatrioside; aralia-saponin V–IV	[195]
		*Coptosapelta flavescens*	*Anthraquinones*: 1,4-dimethoxy-2-methylanthraquinone; 2-amino-3-methoxycarbonyl-1,4-naphtoquinone	[196]
		*Duroia hirsuta*	*Iridoid*: plumericin	[197]
			*Iridoid lactone*: duroin *Flavonol*: ether flavonol-3-*O*-methyl	[198]
		*Duroia macrophylla*	*Triterpenes*: oleanolic acid; ursolic acid	[199]
		*Gardenia collinsae*	*Triterpenes*: 20*R*,24*R*-epoxy-3-oxodammarane-25ξ, 26-diol; C-24-epimer; 20*R*,24*R*-ocotilone	[200]
		*Gardenia gummifera*	*Cycloartane triterpenes:* dikamaliartanes A–F *Flavonoid:* 3′,5,5′-trihydroxy-4′,6,7,8-tetramethoxyflavone	[201]
		*Gardenia jasminoides* **	*Coumarines*: ferrulic acid; skimmin; uracil; 5,8-di-(3-methyl-2,3-dihydroxy-butyloxypsoralen); 3-*O*-α-d-glucopyranosyl-(1→4)-β-d-glucopyranosyloxypeucedanin	[202]
			*Iridoids:* genipin 1-*O*-β-d-d-isomaltoside; 1,10-di-*O*-β-d-glucopyranoside; genipin 1-*O*-β-d-gentiobioside; geniposide; scandoside methyl ester; deacetylasperulosidic acid methyl ester; 6-*O*-methyldeacetylasperulosidic acid methyl ester; gardenoside	[59]
			*Iridoids*: 8-epi-apodantheroside; 7β,8β-epoxy-8α-dihydrogeniposide	[203]
			*Iridoids*: 6′-*O*-[(*E*)-sinapoyl] gardoside; 4′′-*O*-[(*E*)-*p*-coumaroyl]-gentiobiosylgenipin; 6′-*O*-[(*E*)-caffeoyl]-deacetylasperulosidic acid methyl ester	[204]
			*Iridoid*: 6-*O*-sinapoylgeniposide	[205]
			*Monoterpenes*: gardenone; gardendiol	[206]
			*Carotenoids*: crocetin; crocetin mono (β-d-glucosyl) ester; crocetin di-(β-d-glucosyl) ester; crocetin mono-(β-gentiobiosyl) ester; crocetin (β-d-glucosyl)-(β-gentiobiosyl) ester; crocin [crocetin-di-(β-gentiobiosyl)ester]; crocetin (β-gentiobiosyl)-(β-neapolitanosyl) ester; crocetin-di-(β-neapolitanosyl) ester	[207]
			*Monoterpenes*: jasminosides J–K; 6′-*O*-*trans*-sinapoyljasminoside B; 6′-*O*-*trans*-sinapoyljasminoside L; jasminosides M–P; jasminoside C; jasminol E; sacranoside B	[208]
			*Flavonoid*: luteolin-7-*O*-β-d-glucopyranoside *Triterpenes:* ursolic acid; oleanolic acid; methyl 3,4-di-*O*-caffeoylquinate; methyl 5-*O*-caffeoyl-3-*O*-sinapoylquinate; methyl 3,5-di-*O*-caffeoyl-4-*O*-(3-hydroxy-3-methyl)glutaroylquinate; methyl 5-*O*-caffeoyl-4-*O*-sinapoylquinate *Glycosides*: 2-methyl-l-erythritol-4-*O*-(6-*O*-*trans*-sinapoyl)-β-d-glucopyranoside; 2-methyl-l-erythritol-1-*O*-(6-*O*-*trans*-sinapoyl)-β-d-glucopyranoside	[209]
			*Iridoids:* 6′-*O*-*trans*-p-coumaroyl geniposidic acid; 11-(6-*O*-*trans*-sinapoyl glucopyranosyl)-gardendiol; 10-(6-*O*-*trans*-sinapoyl glucopyranosyl)gardendiol; 6′′-*O*-*trans*-sinapoylgenipin gentiobioside; 6′′-*O*-*trans*-cinnamoylgenipin gentiobioside; 10-*O*-succinoylgeniposide; 6′-*O*-acetylgeniposide; 6′′-*O*-*trans-p*-coumaroylgenipin gentiobioside	[210]
			*Iridoids*: gardaloside	[211]
			*Iridoids:* garjasmine; dunnisin; α-gardiol; β-gardiol; diffusoside A diffusoside B; genameside C; deacetylasperulosidic acid	[212]
		*Gardenia jasminoides* var*. radicans*	*Iridoid glycoside*: 6′′-*O*-*trans*-feruloylgenipin gentiobioside; 2′-*O*-*trans*-*p*-coumaroylgardoside; 2′-*O*-*trans*-feruloylgardoside	[213]
		*Gardenia lucida*	*Cycloartane triterpenes:* dikamaliartanes A–F *Flavonoid:* 3′,5,5′-trihydroxy-4′,6,7,8-tetramethoxyflavone	[201]
		*Gardenia saxatilis*	*Triterpenes*: lupenone; lupeol; betulinic acid; messagenic acid A; messagenic acid B; oleanolic acid; ursolic acid; acid (27-*O*-feruloyloxybetulinic acid; 27-*O*-*p*-(*Z*)- and 27-*O*-*p*-(*E*)-coumarate esters of betulinic acid and a mixture of uncarinic acid E (27-*O*-*p*-(*E*)-coumaroyloxyoleanolic acid) and 27-*O*-*p*-(*E*)-coumaroyloxyursolic acid	[214]
		*Gardenia sootepensis*	*Sesquiterpene*: sootepdienone	[215]
		*Gardenia thailandica*	*Flavonoids*: 5,7-dihydroxy-7,2′,3′,4′,5′,6′-hexamethoxyflavone; 5,7-dihydroxy-2′,3′,4′,5′,6′-pentamethoxyflavone; 5-hydroxy-7,2′,3′,4′,5′-pentamethoxyflavone; 5,7-dihydroxy-2′,3′,4′,5′-tetramethoxyflavone *Triterpenes*: thailandiol; gardenolic acid; quadrangularic E acid; 3β-hydroxy-5α-cycloart-24(31)-en-28-oic acid	[216]
		*Gardenia fructus*	*Iridoids*: genipin 1-*O*-β-gentiobioside; 10-*O*-acetylgeniposide; 6α-hydroxygeniposide; 6β-hydroxygeniposide; gardenoside; picrocrocinic acid; 6′-*O*-sinapoyljasminoside; 10-*O*-(4′′-*O*-methylsuccinoyl) geniposide; jasminosides Q–R; 6-*O*-p-coumaroylgeniposide; 6′-*O*-acetylgeniposide; 6′-*O*-sinapoylgeniposide	[217]
			*Iridoids*: geniposidic acid; genipin 1-β-gentiobioside; geniposide; genipin *Flavonoids*: rutin; crocin-1; crocin-2 *Phenolic compound*: chlorogenic acid	[218]
			*Iridoid glycosides*: gardenoside; genipin 1-*O*-β-d-isomaltoside; genipin 1,10-di-*O*-β-d-glucopyranoside; genipin 1-*O*-β-d-gentiobioside; geniposide; scandoside methyl ester; deacetylasperulosidic acid methyl ester	[59]
		*Genipa americana*	*Iridoids*: genipaol; genipin; tarenoside; geniposidic acid; geniposide; genamesides A–D; genipin-gentiobioside; gardenoside; gardendiol; shanzhiside	[219]
			*Monoterpenes*: genipacetal; genipic acid; genipinic acid	
		*Genipa spruceana*	*Cycloartane triterpene*: genipatriol	[220]
		*Lamprothamnus zanguebaricus*	*Phenolic acids*: 1-(3-hydroxy-4-methoxy-5-methylphenyl)-ethanone; 1-(3-hydroxy-4-methoxyphenyl)-ethanone	[221]
		*Oxyanthus pallidus*	*Cycloartane glycosides*: pallidiosides A–C *Triterpenes*: oleanolic acid; 3-*O*-β-d-glucopyranosyl-β-sitosterol	[222]
		*Oxyanthus pyriformis*	Cyanogenic glycosides: prunasin; amygdalin	[223]
		*Oxyanthus speciosus*	*Phenolic compounds:* 2-(2-hydroxy)-ethanol-β-d-glucopyranoside	[61]
			*Cyanogenic glycosides*: holocalin	[223]
		*Pavetta owariensis*	*Proanthocyanidins*: pavetannin A1; pavetannin A2; cinnamtannin B1; pavetanninB1; pavetannin B3; pavetannin B5; pavetannin B6	[224]
		*Psydrax livida*	*Phenolic compounds*: psydroside *Monoterpene*: psydrin	[61]
		*Randia dumetorum*	*Iridoid*: 11-methylixoside	[225]
			*Triterpenes:* α-l-arabinosyl(1→3)-β-galactopyranosyl(1→3)-3-β-hydroxyolean-12-en-28-methyloate	[226]
		*Randia Formosa*	*Triterpenes* *glycosides*: randiasaponins I–VII; ilexoside XXVII; ilexoside XXXVII	[227]
		*Randia siamensis*	*Triterpenes*: ursolic acid; pseudoginsenoside-RP 1; pseudoginsenoside-RT 1	[228]
		*Randia spinosa*	*Iridoid glycosides*: randinoside; galioside; deacetylasperulosidic acid methyl ester; scandoside methyl ester; geniposide; gardenoside	[229]
		*Rothmannia macrophylla*	*Iridoids*: macrophylloside	[230]
		*Rothmannia urcelliformis*	*Iridoid*: genipin *Iridoid alcaloidal*: gardenamide A; 4-oxonicotinamide-1-(1′-β-d-ribofuranoside)	[231]
		*Schumanniophyton problematicum*	*Alkaloids:* rohitukine; rohitukine *N*-oxide; flavopiridol	[232]
		*Scyphiphora hydrophyllacea*	*Iridoid*: scyphiphorin A_1_–A_2_; scyphiphorin B_1_–B_2_	[233,234]
		*Tocoyena brasiliensis*	*Triterpene glycosides*: 3-*O*-β-d-quinovopyranosyl quinovic acid; 3-*O*-β-d-glucopyranosyl quinovic acid; 28-*O*-β-glucopyranosyl ester derivative of quinovic acid *Flavonoid*: ramnazin-3-*O*-rutinoside	[235]
		*Tocoyena bullata*	*Iridoid glycoside*: gardenoside	[236]
		*Tocoyena formosa*	*Iridoids*: α-gardiol; β-gardiol; gardenoside	[237]
	IXO	*Enterospermum madagascariensis*	*Sesquiterpenes*: 2-hydroxy-10-*epi*-zonarene; 2,15-dihydroxycalamenene; guaia-4,6-dien-3-one	[238]
		*Enterospermum pruinosum*	*Triterpenes glycosides:* longispinogenin; 3,16-di-*O*-β-d-glucopyranoside; triacetyllongispinogenin; diglucoside	[239]
		*Ixora coccinea* **	*Triterpene*: ursolic acid	[240]
			*Proanthocyanidins*: ixoratannin A-2; epicatechin; procyanidin A2; cinnamtannin B-1 *Flavonoids*: kaempferol-7-*O*-α-l-rhamnoside; kaempferol-3-*O*-α-l-rhamnoside; quercetin-3-*O*-α-l-rhamnopyranoside; kaempferol-3,7-*O*-α-l-dirhamnnoside	[241]
			*Triterpenes*: lupeol; ixorene; 17β-dammara-12,20-diene-3β-ol	[242,243]
			*Fenolic compounds*: 3-*O*-caffeoylquinic acid; 5-*O*-caffeoylquinic acid; catechin; epicatechin; rutin; quercetin; kaempferol; quercetin 3-*O*-glucoside; quercetin 3-*O*-galactoside; kaempferol 7-*O*-glucoside	[244]
	MUS	*Heinsia crinata*	*Triterpene glycosides*: heinsiagenin A-3β-*O*-(β-glucopyranosyl-(1→2)-β-d-glucopyranosyl-(1→6)-[α-l-rhamnpyranosyl-(1→2)]-β-d-glucopyranosyl-(1→2)-β-d-glucopyranoside); heinsiagenin A-3β-O-(α-l-rhamnopynosyl-(1→2)-β-d-glucopyranosyl-(1→2)-β-d-glucopyranoside)	[245]
		*Mussaenda dona aurora*	*Iridoid glycoside:* shanshiside D	[246]
		*Mussaenda erythrophylla*	*Flavonoid*: 5-hydroxy-7,4′-dimethoxyflavones; *Phenolic compounds:* 3-*iso*-cumaryloxycyclopropane-1-oic acid; 4-hydroxy-3-methoxy cinnamic acid	[247]
		*Mussaenda incana*	*Iridolactona*: shanzhilactone *Iridoid glycosides*: barlerin; mussaenoside *Triterpene*: lupeol	[248]
		*Mussaenda macrophylla*	*Iridoid:* 6-*epi*-barlerin	[249]
		*Mussaenda roxburghii*	*Iridoid*: shanzhiol	[250]
		*Mussaenda pubescens*	*Monoterpenes*: mussaenins A–C	[251]
			*Triterpene glycosides*: mussaendosides R-S; 6 α-hydroxygeniposide; 3β-*O*-β-d-glucopyranosyl quinovic acid 28-*O*-β-d-glucopyranosyl ester	[252]
	OCT	*Villaria odorata*	*Alkenoyloxy alkenol:* villarinol	[253]
			*Iridoids:* morindolide; hydrophylin A; hydrophylin B *Sesquiterpene:* vomifoliol	[254]
	PAV	*Pavetta owariensis*	*Proanthocyanidins*: proanthocyanidin A-2; proanthocyanidin A-4; pavetannin A *Flavonoids*: (+)-catechin; (−)-epicatechin; (+)-epicatechin	[224]
		*Tarenna attenuata*	*Iridoids*: tarenninosides A–G	[255]
		*Tarenna gracilipes*	*Cycloartane glycosides*: tareciliosides H–M	[256]
			*Cycloartane glycosides*: tareciliosides A–G	[257]
		*Tarenna madagascariensis*	*Iridoids*: tarennin; gardenoside; geniposidic acid *Phenolic compounds*: *p*-cumaric acid; cafeic acid; chlorogenic acid *Flavonoids*: kaempferol 3-*O*-β-d-glucopyranoside-7-*O*-α-l-rhamnopyranoside; kaempferol 3-*O*-α-l-rhamnopyranoside-7-*O*-α-l-rhamnopyranoside; quercetin 3-*O*-α-l-rhamnopyranoside-7-*O*-α-l-rhamnopyranoside; kaempferol 3-*O*-α-l-(3′′-*O*-acetyl)-rhamnopyranoside-7-*O*-α-l-rhamnopyranoside; kaempferol 3-*O*-α-l-(4′′-*O*-acetyl) rhamnopyranoside-7-*O*-α-l-rhamnopyranoside	[258]
	POS	*Molopanthera paniculata*	*Iridoid glycosides*: barlerin; shanzhiside methyl ester	[259]
	SAB	*Sabicea brasiliensis*	*Phenolic compounds*: 5-*O*-caffeoylquinic acid; 3,5-*O*-dicaffeoylquinic acid; 4,5-*O*-dicaffeoylquinic acid *Coumarine*: scopoletin *Triterpene*: ursolic acid	[260]
		*Sabicea grisea* var. *grisea*	*Steroid*: octacosanol	[261]
			*Coumarine*: scopoletin *Phenolic compounds*: ethyl caffeate; salicylic acid *Steroid*: 3-*O*-β-d-glucopyranosylsitosterol *Triterpene*: vanillic acid	[262]
	VAN	*Canthium berberidifolium*	*Iridoid glycosides*: 6-*O*-β-d-apiofuranosyl-mussaenosidic acid *Phenolic diglycosides*: canthosides A–D	[263]
		*Canthium multiflorum*	*Iridoid*: 6-oxo-genipin; macrophylloside; garjasmine; gardenine; gardenamide; deacetylasperulosidic acid; 6α-hydroxygeniposide; galioside; aitchisonide B *Triterpenes*: vanillic acid 4-*O*-β-d-(6-*O*-benzoylglucopyranoside); oleanolic acid; quinovic acid	[264]
		*Canthium schimperianum*	*Cyanogenic glycoside esterified with an iridoid glycoside*: 2*R*-[(2-methoxybenzoyl-genoposidyl)-5-*O*-β-d-apiofuranosyl-(1→6)-β-glucopyranosyl-oxy]-2-phenyl acetonitrile; oxyanthin	[265]
		*Fadogia agrestis*	*Monoterpene glycosides*: (2*E*,6*Z*)-2,6-dimethyl-8-[(*O*-α-l-rhamnopyranosyl-(1→3)-α-l-rhamnopyranosyl)-oxy]-octadien-1-yl-α-l-rhamnopyranoside; (2*E*,6*Z*)-2,6-dimethyl-8-[(*O*-α-l-rhamnopyranosyl-(1→3)-α-l-rhamnopyranosyl)-oxy]-octadien-1-yl-*O*-β-d-glucopyranosyl-(1→2)-α-l-rhamnopyranoside; (2*E*,6*Z*)-2,6-dimethyl-8-[(*O*-β-d-glucopyranosyl-(12)-α-l-rhamnopyranosyl)-oxy]-octadien-1-yl-*O*-β-d-glucopyranosyl-(1→2)-α-l-rhamnopyranoside; (2*E*,6*Z*)-2,6-dimethyl-8-[(*O*-α-l-rhamnopyranosyl-(1→3)-(2-*O*-((2*E*,6*Z*)-8-hydroxy-2,6-dimethyloctadienoyl)-α-l-rhamnopyranosyl)-(1→3)-α-l-rhamnopyranosyl) oxy]-octadien-1-yl α-l-rhamnopyranoside; (2*E*,6*Z*)-2,6-dimethyl-8-[(*O*-α-l-rhamnopyranosyl-(1→3)-(2-*O*-((2*E*,6*Z*)-8-hydroxy-2,6-dimethyloctadienoyl)-α-l-rhamnopyranosyl)-(1→3)-4-*O*-acetyl-α-l-rhamnopyranosyl) oxy]-octadien-1-yl α-l-rhamnopyranoside; (2*E*,6*Z*)-2,6-dimethyl-8-[(*O*-α-l-rhamnopyranosyl-(1→3)-(2-*O*-((2*E*,6*Z*)-8-hydroxy-2,6-dimethyloctadienoyl)-α-l-rhamnopyranosyl)-(1→3)-α-l-rhamnopyranosyl)-oxy]-octadien-1-yl-*O*-β-d-glucopyranosyl-(1→2)-α-l-rhamnopyranoside	[266]
		*Fadogia ancylantha*	*Triterpene glycosides*: 3-*O*-β-d-glucopyranosyl-3-β-hydroxyolean-12-en-28-oic acid 28-*O*-[*R*-l-rhamnopyranosyl-(1→2)-β-d-glucopyranosyl] ester; 3-*O*-β-d-glucopyranosyl-3-β-hydroxyolean-12-en-28-oic acid 28-*O*-[-d-apiofuranosyl-(1→2)-β-d-glucopyranosyl] ester	[267]
		*Fadogia homblei*	*Coumarine*: scopoletin *Flavones*: luteolin; quercetin-3-*O*-β-d-galactoside *Triterpenes*: lupeol; betulinic acid; 3β-dodecanoyllup-20(29)-en-28-al; lup-20(29)-en-3β-ylhexadecanoate; oleanolic acid; ursolic acid *Lignan*: 4,4′-dihydroxy-3,3′-dimethoxy-7,9′; 7′,9-diepoxylignan-((−)-pinoresinol)	[268]
		*Vangueria spinosa*	*Proanthocyanidin*: (−)-epicatechin-3-*O*-β-glucopyranoside	[269]
	*	*Augusta longifolia*	*Triterpenes*: ursolic acid; acyl lupeol *Coumarin:* scopoletin *Flavonoids*: naringenin; kaempferol; quercetin; myricitrin; rutin	[270]
		*Myrioneuron nutans*	*Alkaloid:* myrobotinol	[271]
		*Wendlandia formosana*	*Iridoid glycosides*: 10-*O*-caffeoyl scandoside methyl ester; 6-methoxy scandoside methyl ester; scandoside methyl ester; methyl deacetyl asperulosidate; 10-*O*-caffeoyl daphylloside *Triterpene*: ursolic acid	[272]
		*Wendlandia tinctoria*	*Iridoid glycosides*: 5-dehydro-8-*epi*-adoxosidic acid; 5-dehydro-8-*epi*-mussaenoside; 10-*O*-dihydroferuloyldeacetyldaphylloside; wendoside; 8-*epi*-mussaenoside	[273,274]
			*Iridoids:* 5-dehydro-8-*epi*-adoxosidic acid; wendoside	[273]
Rubioideae	ARG	*Argostemma yappii*	*Pyrrolidinoindole alkaloid:* (+)-isochimonanthine	[275]
	COU	*Anthocephalus chinensis*	*Seco-iridoid glycoside*: 3′-*O*-caffeoylsweroside; loganine; 8-epikingiside; loganic acid; sweroside *Phenolic apiglycosides*: kelampayosides A–B *Indole alkaloids*: cadambine; strictosidine lactam; 5α-carboxystrictosidine; desoxycordifoline	[276]
		*Coussarea brevicaulis*	*Triterpenes*: 3-*epi*-spathodic acid; coussaric acid; barbinervic acid; scutellaric acid	[277]
		*Coussarea hydrangeifolia*	*Phenylpropanoid glycosides*: 1′-*O*-benzyl-α-l-rhamnopyranosyl-(1′′→6′)-β-d-glucopyranoside; α-l-xylopyranosyl-(4′′→2′)-(3-*O*-β-d-glucopyranosyl)-10-*O*-(*E*)-caffeoyl-β-d-glucopyranoside; 1,6-di-*O*-caffeoyl-β-d-glucopyranoside; 1-*O*-(*E*)-caffeoyl-β-d-glucopyranoside 1-O-(*E*)-feruloyl-β-d-glucopyranoside	[278]
		*Coussarea paniculata*	*Triterpenes*: lupeol; lupeyl acetate; botulin; betulinic acid; 3-*epi*-betulinic acid; 3-*epi*-betulinaldehyde; oleanolic acid; ursolic acid; lup-20(29)-en-3β,25-diol; lup-20(29)-en-11*R*-ol-25,3β-lactone; 3-deoxybetulonic acid	[279]
		*Coussarea platyphylla*	*Triterpenes*: betulonic acid; betulinic acid *Iridoid*: monotropein *Diterpene*: *trans*-phytol	[280]
		*Cruckshanksia pumila*	*Iridoids*: asperuloside; 7-α-methoxysweroside; swertiamarine	[246,281]
		*Heterophyllaea pustulata*	*Anthraquinones*: soranjidiol; soranjidiol-1-methyl ether; rubiadin; rubiadin-1-methyl ether; damnacanthal; damnacanthol	[282]
			*Anthraquinones*: soranjidiol; rubiadin; rubiadin-1-methyl ether	[283]
	KNO	*Knoxia corymbosa*	*Chromone glycosides*: corymbosins K1–K4; noreugenin; undulatoside A	[284]
		*Knoxia valerianoides*	*Anthraquinones*: 2-hydroxymethylknoxiavaledin; 2-ethoxymethylknoxiavaledin; 2-formylknoxiavaledin	[285]
			*Anthraquinones:* lucidin; lucidin-ω-methyl ether; rubiadin; damnacanthol; 1,3,6-trihydroxy-2-methoxymethylanthraquinone; 3,6-dihydroxy-2-hydroxymethyl-9,10-anthraquinone; 1,3,6-trihydroxy-2-hydroxymethyl-9,10-anthraquinone 3-*O*-β-primeveroside; vanillic acid	[286]
		*Pentas bussei*	*Pentacyclic cyclol-type naphthohydroquinone*: eriobrucinol; methyl 5,10-dihydroxy-7-methoxy-1,1,3α-trimethyl-1a,2,3,3a,10c,10d-hexahydro-1*H*-4-oxacyclobuta[cd]-indeno[5,6-*a*]naphthalene-9-carboxylate	[287]
			*Benzochromene*: methyl-5,10-dihydroxy-7-methoxy-3-methyl-3-[4-methyl-3-pentenyl]-3*H*-benzo[*f*]chromene-9-carboxylate	[288]
		*Pentas lanceolata*	*Anthraquinones*: 5,6-dihydroxydamnacanthol; nordamnacanthal ; lucidin-ω-methyl ether; damnacanthol	[289]
			*Iridoid:* tudoside; 13(*R*)-*epi*-gaertneroside; 13(*R*)-*epi*-epoxygaertneroside; (*E*)-uenfoside; (*Z*)-uenfoside	[290]
		*Pentas longiflora*	*Quinones*: pentalongin; mollugin	[291]
			*Quinones*: pentalongin; mollugin; *trans*-3,4-dihydroxy-3,4-dihydromollugin; methyl-2,3-epoxy-3-prenyl-1,4-naphthoquinone-2-carboxylate; tectoquinone; 3-hydroxymollugin	[289]
		*Pentas micrantha*	*Anthraquinones*: tectoquinone; lucidin-ω-methyl ether; damnacanthol; rubiadin-1-methyl ether; rubiadin; damnacanthal; 5,6-dihydroxydamnacanthol; munjistin methyl ester	[292]
		*Pentas schimperi*	*Anthraquinones*: schimperiquinones A–B; cleomiscosin A; 2-hydroxymethylanthraquinone *Triterpene*: oleanolic acid	[293]
			*Triterpenes*: oleanolic acid; ursolic acid	[294]
	LAS	*Lasianthus fordii*	*Iridoid glycosides*: asperuloside; deacetylasperuloside; methyl deacetyl-asperuloside; megastigmane glucoside; lasianthionoside A–C	[295]
		*Lasianthus gardneri*	*Triterpenes*: lupenone; lupeol; ursolic acid; canaric acid; 3,4-*seco-*lupane	[296]
		*Lasianthus wallichii*	*Iridoids:* iridolactone; iridoid dimer of asperuloside; asperulosidic acid	[297]
		*Ronabea emetic*	*Iridoid glycosides:* asperuloside; 6-hydroxygeniposide; deacetylasperulosidic acid; asperulosidic acid	[298]
	MOR	*Coelospermum billardieri*	*Iridoids:* coelobillardin	[299]
		*Morinda citrifolia* **	*Anthraquinone glycosides*: digiferruginol-1-methylether-11-*O*-β-gentiobioside; digiferruginol-11-*O*-β-primeveroside; damnacanthol-11-*O*-β-primeveroside; 1-methoxy-2-primeverosyloxymethyl-anthraquinone-3-olate; 1-hydroxy-2-primeverosyloxymethyl-anthraquinone-3-olate; 1-hydroxy-5,6-dimethoxy-2-methyl-7-primeverosyloxyanthraquinone	[300]
			*Anthraquinones*: alizarin or 1,2-dihydroxyanthraquinone	[301]
			*Anthraquinones*: 5,15-dimethylmorindol; alizarin 1-methyl ether; anthragallol 1,3-dimethyl ether; anthragallol 2-dimethyl ether; 6-hydroxy-anthragallol-1,3-dimethyl ether; demorindone-5-dimethylether *Iridoids*: morindacin; asuperlosidic acid; deacetylasperulosidic acid	[302]
			*Fatty acid glucosides:* 1,6-di-*O-*octanoyl-β-d-glicopiranose; 6-*O*-(-β-d-glucopyranosyl)-1-*O*-decanoyl-β-d-glicopyranose	[303]
			*Iridoid glycosides*: 6*R*-hydroxyadoxoside; 6β,7β-epoxy-8-*epi*-splendoside; americanin A; narcissoside; asperuloside; asperulosidic acid; borreriagenin; citrifolinin B epimer a; citrifolinin B epimer b; cytidine; deacetylasperuloside; dehydromethoxygaertneroside; *epi*-dihydrocornin; methyl*R*-d-fructofuranoside; methyl-β-d-fructofuranoside; nicotifloroside *Fatty acid* *glycoside* *:* β-sitosterol 3-*O*-β-d-glucopyranoside	[304]
			*Iridoid glycosides*: 9-*epi*-6α-methoxy geniposidic acid	[305]
			*Iridoids:* morindacin	[302]
			*Triterpenes*: 1-*O*-(3′-methylbut-3′-enyl)-β-d-glucopyranose; 1-*n*-butyl-4-(5′-formyl-2′-furanyl)methylsuccinate; 4-*epi*-borreriagenin *Iridoid glycosides*: asperulosidic acid; deacetylasperulosidic acid; 1-*n*-butyl-4-methyl-2-hydroxysuccinate; 1-*n*-butyl-4-methyl-3-hydroxysuccinate	[306]
			*Iridoid glycoside:* citrifoside	[307]
		*Morinda coreia*	*Iridoid glycosides*: yopaaosides A–C; 10-*O*-acetylmonotropein; 6-*O*-acetylscandoside *Phenolic glycosides*: 3,4,5-trimethoxyphenyl 1-*O*-β-apiofuranosyl (1′→6′′)-β-glucopyranoside	[308]
		*Morinda elliptica*	*Anthraquinones*: 2-formyl-1-hydroxyanthraquinone; 1-hydroxy-2-methylanthraquinone; nordamnacanthal; damnacanthal; lucidin-ω-methyl ether; rubiadin; soranjidiol; morindone; rubiadin-l-methyl ether; alizarin-l-methyl ether; morindone-5-methyl ether	[309,310,311]
		*Morinda longissima*	*Coumarine*: scopoletin	[312]
		*Morinda lucida*	*Anthraquinones*: oruwal; oruwalol; damnacanthal; *nor*-damnacanthal; soranjidiol; alizarin-l-methyl ether; rubiadin; rubiadin-l-methyl ether; 2-methylanthraquinone; anthraquinone-2-aldehyde; l-hydroxy-2-methylanthraquinone; l-methoxy-2-methyl-anthraquinone; hexacosanoic acid	[313]
		*Morinda morindoides*	*Flavonoids*: quercetin; quercetin 7,4'-dimethylether; luteolin 7-glucoside; apigenin 7-glucoside; quercetin 3-rhamnoside; kaempferol 3-rhamnoside; quercetin 3-rutinoside; kaempferol 3-rutinoside; chrysoeriol 7-neohesperidoside	[314]
			*Flavonoids*: quercetin; quercetin-3-*O*-rutinoside; kaempferol-7-*O*-rhamnosylsophoroside; chrysoeriol-7-*O*-neohesperidoside; quercetin-7,4′-dimethylether; quercetin-3-*O*-rhamnoside; kaempferol-3-*O*-rhamnoside; kaempferol-3-*O*-rutinoside; apigenin-7-*O*-glucoside; luteolin-7-*O*-glucoside; kaempferol; apigenin; luteolin *Iridoids*: epoxygaertneroside; methoxygaertneroside; gaertneroside; gaertneric acid	[315]
			*Iridoid*: 6′-*O*-acetyl-3′′-methoxygaertneroside	[316]
		*Morinda officinalis*	*Monoterpene*: monotropein	[317]
			*Anthraquinones*: 1,3,8-trihydroxy-2-methoxy anthraquinone; 2-hydroxy-1-methoxy-anthraquinone; rubiadin	[318]
		*Morinda pandurifolia*	*Anthraquinones*: soranjidiol; lucidin-ω-methyl ether; damnacanthal; 1-methoxy-2-methyl anthraquinone; 3-hydroxy-1-methoxy-2-methoxymethyl anthraquinone; anthragallol; nordamnacanthal; flavopurpurin; damnacanthal; lucidin; soranjidiol *Iridoid* *glycoside*: asperulosidic acid	[319]
		*Morinda royoc*	*Anthraquinones:* nordamnacanthal; damnacanthal; lucidin; soranjidiol; rubiadin 1-methylether	[320]
		*Morinda umbellata*	*nor-Iridoids*: umbellatolides A–B	[321]
	OPH	*Lerchea bracteata*	*Alkaloids*: dihydrocorynantheol; dihydrositsirikine; β-hunterburnin methoclhoride; α-hunterburnine methoclhoride; dihydrocorynantheol; melinonine B; methobromide; yombine methobromide; 4-methylanthirine; diploceline; malindine; iso-malindine; dihydro-3-*epi*-corynantheol methoclhoride (lercheine)	[322]
		*Myrioneuron faberi*	*Alkaloid*: myriberine A	[323]
		*Ophiorrhiza blumeana*	*Indole alkaloids*: bracteatine; ophiorrhizine; ophiorrhizine-12-carboxylate; cinchonamine	[324]
		*Ophiorrhiza bracteata*	*Indole alkaloids:* bracteatine	[325]
		*Ophiorrhiza communis*	*Indole alkaloids*: harman; strictosidinic acid	[326]
		*Ophiorrhiza hayatana*	*Anthraquinones*: ophiohayatones A–C	[327]
		*Ophiorrhiza kunstleri*	*Indole alkaloids*: ophiorrhines A–B	[328]
		*Ophiorrhiza liukiuensis*	*Monoterpene glycosides*: demethylsecologanol; 3-*O*-glucosylsenburiside II *Indole alkaloids*: camptothecin; 9-methoxycamptothecin; pumiloside; (3*R*)-deoxypumiloside; 10-methoxycamptothecin; estrictosamide; lyalosidic acid; ophiorrhines A–B; harman *Iridoids*: loganic acid; loganin; swertiaside A *Triterpene*: ursolic acid*;* *epi*-vogeloside *Monoterpene*: sweroside *Flavonoid*: hyperin *Coumarin*: scopoletin	[329]
			*β-Carbolinic alkaloids*: lyalosidic acid; lyaloside; 10-hydroxylyalosidic acid; ophiorrhines A–B; ophiorrhines methyl ester A–B	[330]
		*Ophiorrhiza japonica*	*β-Carbolinic alkaloids*: lyaloside; lyalosidic acid; 10-hydroxylyalosidic acid; ophiorrhines A–B; ophiorrhines methyl ester A–B	
		*Ophiorrhiza pumila* **	*Pentacyclic alkaloid*: camptothecin	[331]
			*Anthraquinones*:1-hydroxy-2-methylanthraquinone; 3-hydroxy-2-methylanthraquinone; 3-hydroxyanthraquinone-2-carbaldehyde; 1-hydroxy-2-hydroxymethylanthraquinone; 3-hydroxy-2-hydroxymethylanthraquinone; 1,3-dihydroxy-2-methylanthraquinone	[332]
			*Alkaloids*: camptothecin; 9-methoxycamptothecin; pumiloside; (3*R*)-deoxypumiloside	[329]
			*Alkaloids*: camptothecin; (3*S*)-pumiloside; (3*S*)-deoxypumiloside; (3*R*)-deoxy-pumiloside; strictosamide	[333]
			*Alkaloids*: camptothecin; pumiloside; (3*S*)-deoxypumiloside; (3*R*)-deoxypumiloside; strictosamide 9-methoxycamptothecin	[330]
		*Ophiorrhiza rosacea*	*Indole alkaloids*: ophiorrhines A and B	[328]
		*Ophiorrhiza rugosa var decumbens*	*Anthraquinones*: 1-hydroxy-2-hydroxymethyl-3-methoxyanthraquinone; 2-*n*-butoxy-methyl-1,3-dihydroxyanthraquinone	[334]
		*Ophiorrhiza trichocarpon*	*Indole alkaloids*: ophiorrhisides A–F; 3,4,5,6-tetradehydrodolichantoside; lyaloside; dolichantoside; 5-oxostrictosidine	[335]
		*Ophiorrhiza tomentosa*	*Indole alkaloids*: harman; strictosidinic acid	[326]
	PAE	*Paederia foetidae*	*Phenolic acid*: ethyl *p*-methoxy-*trans*-cinnamate	[336]
		*Paederia scandens*	*Iridoid glycosides*: paederoside; paederoside B; asperuloside; paederosidic acid; methylpaederosidate; saprosmoside E	[337]
			*Iridoid glycosides*: paederoside; asperuloside; paederosidic acid; asperulosidic acid; paederosidic acid methyl ester; geniposide	[338]
			*Iridoid glycosides*: paederosidic acid; paederoside; asperulosidic acid; asperuloside; geniposidic acid; deacetylasperulosidic acid; decatilasperuloside methyl ester	[339]
			*Iridoid:* 6β-*O*-β-d glucosylparderosic acid	[340]
			*Iridoid glycosides*: asperuloside; paederoside; scanderoside	[341,342]
			*Iridoid* *glycosides*: 6′-*O*-*E*-feruloyl monotropein; 10-*O*-*E*-feruloyl monotropein	[343]
			*Iridoid glycoside:* paederoside B	[344]
	PRI	*Rennellia elliptica*	*Anthraquinone:* 1,2-dimethoxy-6-methyl-9,10-anthraquinone; 1-hydroxy-2-methoxy-6-methyl-9,10-anthraquinone; nordamnacanthal; 2-formyl-3-hydroxy-9,10-anthraquinone; damnacanthal; lucidin-*ω*-methyl ether; 3-hydroxy-2-methyl-9,10-anthraquinone; rubiadin; 3-hydroxy-2-methoxy-6-methyl-9,10-anthraquinone; rubiadin-1-methyl ether; 3-hydroxy-2-hydroxymethyl-9,10-anthraquinone	[345]
	PSY	*Camptotheca acuminata*	*Alkaloids*: camptothecin; 10-hydroxycamptothecin	[346]
		*Carapichea affinis*	*Alkaloids*: cephaeline; emetine; ipecoside; 6-*O*-methylipecoside; 6-*O*-methyl-*trans*-cephaeloside; borucoside	[347]
		*Cephaelis acuminata*	*Alkaloids*: 2-*O*-β-d-glucopyranosyldemethylalangiside; demethylalangiside; 6′′-*O*-β-d-glucopyranosylipecoside; 6′′-*O*-α-d-glucopyranosylipecoside; ipecoside; (4*R*)-4-hydroxy-6,7-di-*O*-methyl ipecoside; (4*S*)-4-hydroxy-6,7-di-*O*-methylipecoside; 6,7-di-*O*-methylipecoside tetraacetate	[348]
			*Alkaloids*: emetine; cephaeline; neocephaeline 7-*O*-demethylcephaeline; 10-*O*-demethylcephaeline; 2′-*n*-(1′′-deoxy-1′′-β-d-buctopyranosyl) cephaeline; 2′′-*n*-(1′′-deoxy-1′′-β-d-fructopyranosyl) pyranosyl	[349]
		*Cephaelis acuminata*	*Alkaloids*: neocephaeline; 7′-*O*-demethylcephaeline; 10-*O*-demethylcephaeline; 2′-*n*-(10-deoxy-10-β-d-fructopyranosyl) cephaeline; 2′-*n*-(10-deoxy-10′′-β-d-fructopyranosyl) neocephaeline; emetine; cephaeline; psychotrine; protoemetine; 9-demethylprotoemetinol; isocephaeline	[349]
		*Cephaelis dichroa*	*Indole alkaloids*: vallesiachotamine lactone; vallesiachotamine; strictosamide; strictosidine; angustine	[350]
		*Cephaelis ipecacuanha*	*Tetrahydroisoquinoline-monoterpene glucosides:* 3-*O*-demethyl-2-*O*-methylalangiside; alangiside or ipecoside; 6-*O*-methylipecoside; 7-*O*-methylipecoside; 3-*O*-demethyl-2-*O*-methylalangiside; 2-*O*-methylalangiside	[351]
			*Alkaloids*: emetine; cephaeline; psychotrine; emetamine; *O*-methylpsycotrine	[352]
		*Chassalia curviflora* var. *ophioxyloides*	*Indole alkaloids:* alstrostine A; rudgeifoline	[353]
		*Margaritopsis cymuligera*	*Pyrrolidinoindoline alkaloids*: hodgkinsine; quadrigemine C	[354]
		*Palicourea acuminata*	*Indole alkaloid*: strictosidinic acid; methylester strictosidine; palicoside; bahienoside B; 5α-carboxystrictosidine; desoxycordifoline; lagamboside; vallesiachotamine	[355]
		*Palicourea adusta*	*Monoterpenoid glucoindole alkaloids*: lyaloside; tetra-(*O*-acetyl)-lyaloside; (*E*)-*O*-(6′)-cinnamoyl-4′′-hydroxy-3′′-methoxylyaloside; (*E*)-tetra-(*O*-acetyl)-*O*-(6′)-cinnamoyl-4′-hydroxy-3′-methoxylyaloside; (*E*)-tetra-(*O*-acetyl)-*O*-(6′)-cinnamoyl-4′′-hydroxy-3′′,5′′-dimethoxylyaloside	[356]
		*Palicourea crocea*	*Monoterpenoid indole alkaloids:* 3,4-dihydro-1-(1-β-d-glucopyranosyloxy-1,4α,5,7-tetrahydro-4-methoxycarbonylcyclopenta[c]pyran-7-yl)-β-carboline-N2-oxide; croceaine A; psychollatine	[357]
		*Palicourea coriacea*	*Glucoindole alkaloids*: 3-*epi*-strictosidinic acid; strictosidinic acid; strictosidinic ketone *Alkaloid*: calycanthine *Triterpene*: ursolic acid	[358]
		*Palicourea crocea*	*Monoterpene Indole Alkaloids*: croceaines A–B	[359]
		*Palicourea rigida*	*Indole alkaloid*: vallesiachotamine	[360]
		*Prismatomeris connata*	*Anthraquinone glycosides*: 1-*O*-methylrubiadin 3-*O*-β-primeveroside; damnacanthol 3-*O*-β-primeveroside; rubiadin 3-*O*-β-primerveroside; lucidin 3-*O*-β-primeverosideo; 1,3-dihydroxy-2-(methoxymethyl) anthraquinone 3-*O*-β-primerveroside; digiferruginol ω-gentiobiose	[361]
			*Phenolic compound glycoside*: prismaconnatoside	[362]
		*Prismatomeris malayana*	*Anthraquinone:* 1,3-dihydroxy-5,6-dimethoxy-2-methoxymethyl-9,10-anthraquinone; 2-hydroxymethyl-1-methoxy-9,10-anthraquinone; tectoquinone; 1-hydroxy-2-methyl-9,10-anthraquinone; rubiadin; rubiadin-1-methyl ether; 1,3-dihydroxy-5,6-dimethoxy-2-methyl-9,10-anthraquinone; nordamnacanthal; damnacanthal	[363]
		*Prismatomeris tetrandra*	*Iridoids:* prismatomerin	[364,365]
		*Psychotria bahiensis*	*Bis(monoterpenoid) indole alkaloid glucosides:* bahienoside A; bahienoside B; 5R-carboxystrictosidine; angustine; strictosamide; (*E*)- and (*Z*)-vallesiachotamine	[366]
		*Psychotria barbiflora*	*β-Carbolinic* *alkaloids*: harman; strictosidinic acid	[367]
		*Psychotria brachyceras*	*Monoterpene indole alkaloids*: brachycerine	[368]
		*Psychotria camponutans*	*Pyranonaphthoquinones*: pentalongin; psychorubrin; 1-hydroxy-3,4-dihydro-1*H*-benz[g]isochromene-5,10-dione	[369]
		*Psychotria colorata*	*Alkaloids*: (−)-calycanthine; isocalycanthine; (+)-chimonanthine; hodgkinsine; quadrigemine C; (8-8a),(8′-8′a)-tetradehydroisocalycanthine 3a(*R*),3′a(*R*)	[370]
		*Psychotria calocarpa*	*Alkaloids*: psychotriasine	[371]
		*Psychotria correae*	*Indole alkaloids*: isodolichantoside; correantoside; 10-hydroxycorreantoside; correantines A–C e 20-*epi*-correantine B *C13-Norisoprenoids*: megastigm-5-ene-3,9-diol; *S*(+)-dehydrovomifoliol *Carotenoids*: lutein	[372]
		*Psychotria glomerulata*	*Quinoline alkaloids*: glomerulatines A−C; calycanthine; *iso*-calycanthine	[373]
		*Psychotria ipecacuanha*	*Alkaloids*: emetine; cephaeline	[374]
		*Psychotria leiocarpa*	*Indole alkaloids*: umbellatine; brachicerine; lyaloside; strictosamide; myrianthosines A–B; *n*,β-D-glucopyranosyl vincosamide quadrigemine A *Iridoid glucosides*: asperuloside; deacetylasperuloside; loganin	[375]
		*Psychotria myriantha*	*Indole alkaloids*: strictosidinic acid	[376]
			*Indole alkaloids*: strictosidinic acid	[377]
		*Psychotria nuda*	*Alkaloid:* strictosamide	[378]
		*Psychotria lyciiflora*	*Alkaloids: meso*-chimonanthine; hodgkinsine; *N*-demethyl-meso- chimonanthine; quadrigemine C; isopsycotridine B; psychotridine; quadrigemine I; oleoidine; caledonine	[379]
		*Psychotria oleoides*		
		*Psychotria prunifolia*	*Alkaloids:* strictosamide; 10-hydroxyiso-deppeaninol; *N*-oxide-10-hydroxy-antirhine	[380]
			*Indole-β-carboline alkaloids:* 10-hydroxyisodeppeaninol; *N*-oxide-10-hydroxy-antirhine; 14-oxoprunifoleine; strictosamide	[381]
			*Indole-β-carboline alkaloids:* 14-oxoprunifoleine; strictosamide; 10-hydroxyantirhine N-oxide; 10-hydroxyisodeppeaninol	[382]
		*Psychotria suterella*	*Indole alkaloids*: lyaloside; naucletine; strictosamide	[383]
		*Psychotria umbellata*	*Indole alkaloids*: psycollatine	[384]
		*Psychotria vellosiana*	*Triterpenes*: squalene; lupeolids *Coumarin*: scopoletin	[385]
		*Psychotria viridis*	*Alkaloid*: dimethyltryptamine	[386]
		*Rudgea jasminoides*	*Anthraquinone*: 1,4-naphthohydroquinone	[387]
	PUT	*Plocama pendula*	*Naphthohydroquinones*: mollugin 6-methyl ether; plocanaphthin *Lignans:* syringaresinol; pinoresinol; lariciresinol *Coumarin:* scopoletin	[388]
			*Anthraquinones*: balonone; balonone; methyl ether; plocamanones A–C; knoxiadin; 5,6-dimethyl ether; plocamanone D; chionone; isozyganein dimethyl ether; lucidin 1,3-dimethyl ether; lucidin; 1-hydroxy-2-methyl-9,10-anthraquinone; tectoquinone; rubiadin 3-methyl ether; rubiadin 1-methyl ether; rubiadin dimethyl ether; rubiadin; lucidin 3-methyl ether; munjistin ethyl ester; ibericin; damnacanthol ω-ethyl ether; alizarin dimethyl ether; alizarin 1-methyl ether; anthragallol 1,2-dimethyl ether; 3-hydroxy-2-(hydroxymethyl)-9,10-anthraquinone	[389]
			*Triterpenes*: 3-*epi*-pomolic acid 3α-acetate; baloic acid; meth; 19α-hydroxyoleanonic acid; 3β-hydroxyolean-11,13(18)-dien-28-oic acid; 3α-acetoxy-19α-hydroxyursa-12-en-28-oic acid; baloic acid;19α-hydroxyoleanonic acid	[390]
		*Putoria calabrica*	*Flavonoids*: calabricosides A–B *Iridoid*: asperuloside; paederosidic acid; paederoside *Lignan glycosides*: liriodendrin; dihydrodehydrodiconiferyl alcohol-4-*O*-β-d-glucopyranoside; 7*S*,8*R*,8′*R*-(–)-lariciresinol-4,4′-bis-*O*-β-d-glucopyranoside.	[391]
	SPE	*Borreria verticillata*	*Indole alkaloids*: spermacoceine; borrerine; borreverine; isoborreverine	[392]
			*Indole alkaloids*: verticillatines A–B *Iridoids*: scandoside methyl ester; 6′-*O*-(2-glyceryl) scandoside methyl ester; asperuloside acid	[393]
		*Dunnia sinensis*	*Iridoid*: dunnisinine *Iridoid glycoside*: dunnisinoside	[394]
		*Galianthe brasiliensis*	*Iridoid glycosides*: asperuloside; deacetylasperuloside; mixture of *Z*- and *E*-6-*O*-*p*-coumaroylscandoside methyl ester	[395]
		*Galianthe ramosa* **	*Phenolic compound*: epicatechin *Triterpene*: ursolic acid *β-carboline indole alkaloid*: 1-(hydroxymethyl)-3-(2-hydroxypropan-2-yl)-2-(5-methoxy-9*H*-β-carbolin-1-yl) cyclopentanol	[396]
			*β-carboline alkaloid*: 1-(hydroxymethyl)-3-(2-hydroxypropan-2-yl)-2-(5-methoxy-9*H*-β-carbolin-1-yl) cyclopentanol; 9-methoxyindole alkaloid	[396]
		*Galianthe thalictroides*	*β-carboline indole alkaloid*: 1-methyl-3-(2-hydroxypropan-2-yl)-2-(5-methoxy-9*H*-β-carbolin-1-yl)-cyclopentanol; 1-(hydroxymethyl)-3-(2-hydroxypropan-2-yl)-2-(5-methoxy-9*H*-β-carbolin-1-yl)-cyclopentanol *Anthraquinones:* 1-methylalizarin; morindaparvin-A *Coumarin*: scopoletin	[397]
		*Hedyotis auricularia*	*β-Carboline alkaloid*: auricularine	[398]
		*Hedyotis capitellata*	*β-Carboline alkaloids*: capitelline; cyclocapitelline; isocyclocapitelline; hedyocapitelline; hedyocapitine	
		*Hedyotis chrysotricha*	*β-Carboline alkaloid*: chrysotricine	
		*Hedyotis capitellata*	*Anthraquinones*: capitellataquinone A–D; rubiadin; anthragallol; 2-methyl ether; alizarin-1-methyl eter; digiferruginol; lucidin-3-*O*-β-glucoside	[399]
			*β-Carboline alkaloids*: capitelline; (−)-isocyclocapitelline; (+)-cyclocapitelline; isochrysotricine; chrysotricine	[400]
			*β -Carboline alkaloids*: capitelline; (+)-isocyclocapitelline; (+)-cyclocapitelline; isochrysotricine; chrysotricine	[401]
		*Hedyotis chrysotricha*	*β-Carboline alkaloid*: chrysotricine	[402]
		*Hedyotis corymbosa*	*Iridoid glucosides*: asperuloside; scandoside methyl ester	[403]
			*Iridoids:* hedycoryside A–C	[404]
		*Hedyotis crassifolia*	*Triterpenes*: ursolic acid; 3β-hydroxyurs-11-ene-23(13)-lactone; 3α,13β-dihydroxyurs-11-ene-28-oic acid; oleanolic acid; 3-β-d-glucopyranosyl-β-sitosterol and 3β,6β-dihydroxyolean-12-ene-28-oic acid	[405]
		*Hedyotis diffusa* **	*Iridoid glycosides*: dunnisinoside; *E*-6-*O*-*p*-methoxycinnamoyl scandoside methyl ester; *Z*-6-*O*-*p*-methoxycinnamoyl scandoside methyl ester; *E*-6-*O*-*p*-feruloyl scandoside methyl ester; *E*-6-*O*-*p*-coumaroyl scandoside methyl ester; *Z*-6-*O*-*p*-coumaroyl scandoside methyl ester	[406]
			*Iridoid* *glucosides:* diffusosides A–B	[407]
			*Anthraquinones*: 2-methyl-3-methoxyanthraquinone; 2-methyl-3-hydroxyanthraquinone; 2-methyl-3-hydroxy-4-methoxyanthraquinone; 2,3-dimethoxy-6-methylanthraquinone	[398]
			*Flavonoids*: quercetin; quercetin 3-*O*-glucopyranoside; quercetin 3-*O*-sambubioside; quercetin 3-*O-*sophoroside; quercetin 3-*O*-rutinoside	
		*Hedyotis dichotoma*	*Anthraquinones*:1,4-dihydroxy-2,3-dimethoxyanthraquinone; 1,4-dihydroxy-2-hydroxy-methylanthraquinone; 2,3-dimethoxy-9-hydroxy-1,4-anthraquinone; 2-hydroxymethyl-10-hydroxy-1,4-anthraquinone *Flavonoids*: isovitexin	[398]
		*Hedyotis intricata*	*Triterpene*: lupeol; oleanolic acid *Iridoid:* asperuloside	[408]
		*Hedyotis hedyotidea*	*Iridoids*: deacetylasperulosidic acid ethyl ester; hedyotoside; asperulosidic acid; asperuloside; deacetylasperuloside	[409]
		*Hedyotis herbacea*	*Flavonoids*: kaempferol 3-*O*-rutinoside; rutin; kaempferol 3-*O*-glucoside; kaempferol 3-*O*-arabinopyranoside; kaempferol-3-*O*-arabino pyranoside; quercetin 3-*O*-galactoside	[398,410] [410]
		*Hedyotis nudicaulis*	*Triterpene glycosides*: nudicaucins A–C; guaiacin D	[411]
		*Hedyotis pinifolia*	*Anthraquinones*:1,6-dihydroxy-7-methoxy-2-methylanthraquinone; 1,6-dihydroxy-2-methylanthraquinone; 3,6-dihydroxy-2-methylanthraquinon; 1,3,6-trihydroxy-2-methylanthraquinone	[412]
		*Hedyotis tenelliflora*	*Iridoids:* teneoside B	[413]
		*Hedyotis verticillata*	*Flavonoids*: kaempferitrin	[398]
		*Hedyotis vestita*	*Stereoid*: phytol *Flavonoids:* rutine; isohrametin 3-*O*-rutinoside; vomifoliol 9-*O*-β-d-glucopyranoside; auricularin *Iridoid:* 6α-methoxygenyposide; *Phenolic compound:* sodium (1*S*,4a*R*,5*R*,7a*R*)-7-hydroxymethyl-5-methoxy-1-β-d-glucopyranosyloxy-1,4α,5,7α-tetrahydrocyclopenta[c]pyran-4-carboxylate	[414]
		*Mitracarpus frigidus*	*Pyranonaphthoquinone*: psychorubrin	[415]
		*Mitracarpus scaber* **	*Pentalongin hydroquinone diglycoside*: harounoside	[416]
			*Phenolic compounds*: pentadecanoic; (*Z*)-octadec-9-enoic; tetradecanoic; (*Z*,*Z*)-octadeca-9,12-dienoic; (*Z*)-hexadec-9-enoic; octadecanoic; dodecanoic acid	[417]
		*Mitracarpus villosus*	*Triterpenes*: methyl ursalate; ursolic acid	[418]
		*Oldenlandia corymbosa*	*Iridoid glycosides*: geniposidic acid; scandoside; feretoside; 10-*O*-benzoylscandoside methyl ester; odenlandoside III; asperulosidic acid; deacetylasperulosidic acid	[419]
		*Oldenlandia difusa*	*Triterpenes*: ursolic acid	[420]
			*Triterpenes*: 2,6-dihydroxy-1-methoxy-3-methylanthraquinone; 2-hydroxy-1-methoxy-3-methylanthraquinone; 2-hydroxy-3-methylanthraquinone; quercetin-3-*O*-[2-*O*-(6-*O*-E-sinapoyl)-β-d-glucopyranosyl]-β-glucopyranoside; quercetina-3-*O*-[2-*O*-(6-*O*-E-feruloyl)-β-d-glucopyranosyl]-β-glucopyranoside; kaempferol-3-*O*-[2-*O*-(6-*O*-E-feruloyl)-β-d-glucopyranosyl]-β-galactopyranoside; quercetin-3-*O*-(2-*O*-β-d-glucopyranosyl)-β-d-glucopyranoside; rutin; quercertin	[421]
		*Oldenlandia umbellata*	*Anthraquinones*: 1,2,3-trimethoxyanthraquinone; 1,3-dimethoxy-2-hydroxy-anthraquinone; 1,2-dimethoxyanthraquinone; 1-methoxy-2-hydroxyanthraquinone; 1,2-dihydroxyanthraquinone	[422]
		*Richardia grandiflora*	*Phenolic compounds: o*-hydroxybenzoic acid; *m*-methoxy-*p*-hydroxybenzoic acid	[423]
		*Saprosma fragrans*	*Anthraquinones*: 4-dihydroxy-1-methoxyanthraquinone-2-corboxaldehyde; damnacanthal	[424]
		*Saprosma hainanense*	*Alkaloids*: saprosmine A; saprosmine B; marcanine A; quinolone; cleistopholine; 4-methoxycarbonyl-5; 10-benzogquinolinequinone; liriodenine	[425]
		*Saprosma scortechinii*	*Iridoid:* 6-*O*-*epi*-acetylscandoside	[426]
			*Iridoids:* 10-*O*-benzoyl deacetylasperulosidic acid; 3,4-dihydro-3α-methoxy-paederoside; saprosmosides A–H	[426]
			*Bis-iridoid glucosides*: saprosmosides A–F *Iridoid glucosides:* 3,4-dihydro-3-methoxypaederoside; 10-*O*-benzoyldeacetylasperulosidic acid; deacetylasperuloside; asperuloside; paederoside; deacetylasperulosidic acid; scandoside; asperulosidic acid; 10-acetylscandoside; paederosidic acid; 6-*epi*-paederosidic acid; methylpaederosidate; monotropein	[427]
		*Saprosma ternatum*	*Alkaloid*: vittadinoside *Coumarins:* scopoletin *Iridoid glycosides:* epiasperuloside; epipaederosidic acid; epipaederosi *Triterpenes:* betulinic acid; betulinaldehyde	[428]
		*Spermacoce verticillata*	*Triterpenes*: morolic acid; oleanolic acid; ursolic acid; 3,5-dioxofriedelane *Flavonoids*: 3-*O*-α-l-rhamnopyranosyl quercetin; quercetin *Anthraquinones*: 2-hydroxy-3-methylanthraquinone	[429]
	RUB	*Asperula maximowiczii*	*Iridoids*: asperuloides A–C	[430]
		*Crucianella graeca*	*Coumarins*: daphnin; daphnetin; daphnetin glucoside *Iridoids*: deacetylasperulosidic acid; scandoside; asperuloside; asperulosidic acid; methyl ester of deacetylasperulosidic acid; dafiloside; geniposidic acid; 10-hydroxyloganin; deacetylasperuloside	[431]
		*Crucianella maritima*	*Iridoid*: deacetylasperulosidic acid 6'-glucoside sodium salt; *Anthraquinones*: 1-hydroxy-2-carbomethoxyanthraquinone; 6-methylanthragallol-2-methyl ether; 6-methylanthragallol-2,3-dimethyl ether; 6-methoxy-2-methylquinizarin; 1-hydroxy-2-methyl-6-methoxyanthraquinone	[432]
			*Iridoids*: asperuloside; asperulosidic acid; deacetylasperulosidic acid	[433]
		*Cruciata glabra*	*Coumarins*: daphnin; daphnetin; daphnetin glucoside *Iridoids*: scandoside	[431]
		*Cruciata laevipes*	*Coumarins*: daphnin; daphnetin glucoside *Iridoids*: scandoside; asperuloside; asperulosidic acid; methyl ester of deacetylasperulosidic acid; daphylloside	
		*Cruciata pedemontana*	*Coumarins*: daphnin; daphnetin glucoside *Iridoids*: scandoside; asperuloside; asperulosidic acid; methyl ester of deacetylasperulosidic acid; daphylloside	
		*Cruciata taurica*	*Monoterpenoid glycosides*: cruciaside A (2,5-*O*-β-d-diglucopyranosyl-3-hydroxy-*p*-cymene); cruciaside B (5-*O*-β-d-glucopyranosyl-2,3-dihydroxy-*p*-cymene)	[434]
			*Coumarin glucosides*: daphnin; daphnetin glucoside; 7-*O*-(6′-acetoxy-β-d-glucopyranosyl)-8-hydroxycoumarin; 7-*O*-[6′-*O*-(3′′,4′′-dihydroxycinnamoyl)-β-d-glucopyranosyl]-8-hydroxycoumarin	[435]
		*Crucianella graeca*	*Iridoids*: deacetylasperulosidic acid; scandoside; asperuloside; asperulosidic acid; geniposidic acid; 10-hydroxyloganin; deacetylasperuloside; iridoid V3	[431]
		*Galium album*	*Iridoid glycosides*: secogalioside; asperuloside; deacetyl asperulosidic acid; scandoside; monotropein; asperulosidic acid; geniposidic acid; 10-hydroxyloganin; 10-hydroxymorroniside (isomers 7α e7β); daphylloside	[436]
		*Galium aparine*	*Anthraquinone aldehyde:* nordamnacanthal	[437]
		*Galium lovcense*	*Iridoid glycosides*: secogalioside; asperuloside; deacetyl asperulosidic acid; scandoside; monotropein; asperulosidic acid; geniposidic acid; 10-hydroxyloganin; 10-hydroxymorroniside (isomers 7α e7β); daphylloside; 7-β-hydroxy-11-methyl forsythide; 7-*O*-acetyl-10-acetoxyloganin	[436]
		*Galium rivale*	*Iridoid glycosides:* monotropein; scandoside; eacetylasperulosidic acid; geniposidic acid; asperulosidic acid *Triterpene glycosides*: rivalosides A–E e momordin II	[438]
		*Galium macedonicum*	*Iridoid*: macedonine	[439]
		*Galium sinaicum*	*Anthraquinones*: 6,7-dimethoxyxanthopurpurin; 6-hydroxy-7-methoxyrubiadin; 5-hydroxy-6-hydroxymethyl anthragallol 1,3-dimethyl ether; 7-carboxyanthragallol 1,3-dimethyl ether; anthragallol l-methyl ether 3-*O*-β-d-glucopyranoside; anthragallol l-methyl ether 3-*O*-rutinoside; anthragallol 3-*O*-rutinoside; alizarin 1-methyl ether 2-*O*-primeveroside	[440]
		*Galium spurium*	*Flavonoids*: asperulosidic acid ester ; asperuloside; caffeic acid; kaempferol-3-*O*-l-rhamnopyranoside; quercetin-3-*O*-[α-l-rhamnopyranosyl(1→6)-β-d-glucopyranoside]; isorhamnetin-3-*O*-glucopyranoside; quercetin-3-*O*-α-l-rhamnopyranoside; kaempferol-3-*O*-[α-l-rhamnopyranosyl(1→6)-β-d-glucopyranoside]; quercetin	[441]
		*Galium verum*	*Anthraquinones*: 1,3-dihydroxy-2 methoxy methyl; 1,3-dimethoxy-2-hydroxy; 1,3-dihydroxy-2-acetoxy; 1-hydroxy-2-hydroxy-methyl; 1,3-dihydroxy-2-methyl; 1-methoxy-2-hydroxy; 1,3-dihydroxy-2-hydroxy-methyl-6-methoxy; 1,6-dihydroxy-2-methyl anthraquinones	[442]
		*Galium verum var. asiaticum*	*Iridoid glycoside*: 10-*p*-dihydrocoumaroyl-6-α-hydroxygeniposide; 10-*p*-dihydrocoumaroyl deacetylasperuloside; asperulosidic acid methyl ester; asperuloside; asperulosidic acid; deacetylasperuloside; scandoside	[443]
		*Rubia akane*	*Anthraquinones*: 1,3-dihydroxyanthraquinone-2-al; lucidin-3-*O*-primeveroside	[437]
		*Rubia cordifolia*	*Naphtoquinones*: dihydromollugin; 2-carbomethoxy-3-(3'-hydroxy)-isopentyl-1,4-naphthohydroquinone 1,4-*O*-di-β-glucoside; 2-carbomethoxy-3-(3'-hydroxy) isopentyl-1,4-naphthohydroquinona 4-*O*-β-glucoside *Anthraquinones*: xanthopurpurin; 2-methyl-1,3,6-trihydroxy-9,10-anthraquinone 3-*O*-β-glucoside; 2-methyl-1,3,6-trihydroxy-9,10-anthraquinone; 2-methyl-1-hydroxy-9,10-anthraquinone; 3-*O*-α-rhamnosyl(1→2)-β-glucoside; 3-*O*-(6'-*O*-acetyl)-α-rhamnosyl (1→2)-β-glucoside; 2-methyl-1,3,6-trihydroxy-9,10-anthraquinone 3-*O*-(4′,6′-*O*-diacetyl)-α-rhamnosyl (1→2)-β-glucoside; 2-methyl-1,3,6-trihydroxy-9,10-anthraquinone 3-*O*-(3′,6′-*O*-diacetyl)-α-rhamnosyl (1→2)-β-glucoside	[444]
			*Iridoids glycoside*: 6-methoxygeniposidic acid; 6-methoxygeniposidic acid methyl ester *Triterpene*: oleanolic aldehyde acetate *Fenolic compound*: furomollugin	[445]
		*Rubia peregrina*	*Anthocyanins*: cyanidin 3-*O*-glucoside; delphinidin 3-*O*-glucoside; cyanidin 3-*O*-arabinoside	[446]
		*Rubia schumanniana*	*Anthraquinones glycosides*: 1,3,6-trihydroxy-2-methyl anthraquinone; (2-methyl-1,3,6-trihydroxy-9,10-anthraquinone-3-*O*-α-L-rhamnopyranosyl (1→2)-β-d-glucopyranoside); 1-hydroxy-2-hydroxy-methylene-9,10-anthraquinone-11-*O*-β-d-glucopyranosyl (1→6)-β-d-glucopyranoside; digiferruginol glycoside	[447]
			*Triterpenes:* 3β-hydroxy-urs-30-*p*-*Z*-hydroxycinnamoyl-12-en-28-oic-acid; 3β-hydroxy-olean-30-*p*-*E-*hydroxycinnamoyl-12-en-28-oic-acid; 3β,6α-dihydroxy-urs-14-en-12-one	[448]
			*Cyclopeptides:* rubischumanins A–C; C-6β-oxy-RA IV; RA-IV; *O*-seco-RA-V	[448]
		*Rubia yunnanensis*	*Triterpene:* rubiarbonol K	[449]
		*Rubia tinctorum*	*Anthraquinones*: alizarin; lucidin; mollugin; xanthopurpurin; rubiadin	[450]
			*Anthraquinones*: 1-hydroxy-2-hydroxymethylanthraquinone 3-glucoside 2-hydroxymethyl-anthraquinone 3-glucoside; 3,8-dihydroxymethylanthraquinone 3-glucoside *Anthraquinone glycosides*: alizarin; lucidian-ω-ethyl ether; lucidin primeveroside *Iridoid*: asperuloside	[451]
			*Anthraquinones*: pseudopurpurin; lucidin; alizarin; purpurin; alizarin-2-methylether; lucidin-ω-ethylether; nordamnacanthal; munjistin ethyl ester; lucidin primeveroside; ruberithric acid	[452,453]
		*Rubia yunnanensis*	*Cyclic hexapeptides*: rubiyunnanins A–B	[454]
			*Triterpenes:* rubiarbonones D–F; rubiarbosides F–G; rubiarbonone A; rubiarbonol A–B; rubiarbonone B; rubiarbonol A; rubiarbonol B; rubiarbonol F; rubiarbonol G; rubiarboside A	[455]
**	*	*Luculia pinciana*	*Triterpene:* luculiaoic acid A	[456]
			*Triterpenes:* vogeloside; epi-vogeloside; loganoside; loganin; cincholic acid 28-*O*-β-d-glucopyranosyl ester; cincholic acid-3-*O*-β-d-glucopyranoside, 28-*O*-β-d-glucopyranosyl ester; cincholic acid-3-*O*-β-d-glucopyranoside	[457]

ALB: Alberteae; ARG: Argostemmateae; CHI: Chiococceae; CIN: Cinchoneae; COF: Coffeeae; CON: Condamineeae; COU: Coussareeae; GAR: Gardenieae; GUE: Guettardeae; HAM: Hamelieae; HIL: Hillieae; HYM: Hymenodictyeae; ISE: Isertieae; IXO: Ixoreae; KNO: Knoxieae; LAS: Lasiantheae; MOR: Morindeae; MUS: Mussaendeae; NAU: Naucleeae; OCT: Octotropideae; OPH: Ophiorrhizeae; PAE: Paederieae; PAV: Pavetteae; POS: Posoquerieae; PRI: Prismatomerideae; PSY: Psychotrieae; PUT: Putorieae; RUB: Rubieae; SAB: Sabiceeae; SPE: Spermacoceae; VAN: Vanguerieae. * Genera not allocated to any tribe. ** Genera unclassified to subfamily.

This survey found Rubioideae subfamily has the highest chemical diversity in Rubiaceae subfamily. Among the described tribes, the most chemically studied are: Naucleeae (44), Gardenieae (39), Psycotrieae (34), Spermacoceae (35), Rubieae (25) and Ophiorrhizeae (14); other tribes have around five to six studied species. In general, the species with the largest number of phytochemical studies recorded from 1990 to 2014 belong to the genera *Uncaria*, *Psychotria*, *Hedyotis*, *Ophiorrhiza* and *Morinda*. Plants from the Psycotrieae tribe were shown to be the major producers of alkaloids, since all phytochemical studies with genera belonging to this tribe (*Camptotheca*, *Carapichea*, *Cephaelis*, *Chassalia*, *Margaritopsis*, *Palicourea* and *Psychotria*) resulted in the isolation of alkaloids. In the Gardenieae tribe, the presence of iridoids was observed, not only in this survey, but also in other studies [59,60,61,62,64]. Studies showed *Rubia*, *Galium* and *Morinda* genera (subfamily Rubioideae) as important sources of anthraquinones, such as aglycone and rarely glycosides [56].

However, studies establishing a chemotaxonomic classification of plants are quite complex, since there are different types of secondary metabolites that can be distinct in correlated species. These differences in the production of secondary metabolites can be attributed to a number of factors such as genetic mutation, blocking of a biosynthetic pathway and changes in the metabolism due to infection. Soil and climatic variations such as altitude, soil type, macronutrients, micronutrients and water availability, plant age, ultraviolet radiation, rainfall, seasonality and circadian rhythm, also have great influence on the production of metabolites. Besides the fact that the chemical composition can be variable in accordance with the plant organ, it is necessary to study the plant as a whole, to be able to infer a degree of similarity [59,60,61,62,63,64].

Considering the chemical profile of the Rubiaceae family and the metabolic pathways used to produce it, Rubioideae is the most ancient subfamily from an evolutive point of view [16], then it was subdivided into Ixoroideae and finally into Cinchonoideae. The chemical biosynthetic pathway now supports this botanical conclusion. In Rubioideae, anthraquinones are the main metabolites and the pathways are not so specific, being iridoids and indole alkaloids produced also in a large amount. In Ixoroideae, the most active biosysthetic pathway is the one that produces iridoids; while in Cinchonoideae, it is the one that produces indole alkaloids together with other alkaloids.

## 6. Conclusions

This review has encompassed phytochemical studies of Rubiaceae species for the past 24 years. These substances have been isolated mainly from *Uncaria*, *Psychotria*, *Hedyotis*, *Ophiorrhiza* and *Morinda* genera. From the Rubioideae subfamily, 139 species were studied; 80 from the Ixoroideae, and 74 from the Cinchonoideae. Some correlations between iridoids, triterpenes, alkaloids and anthraquinones occurrence and distribution between tribes and subfamilies could be observed, providing chemotaxonomic clues. From an evolutionary point of view, the Rubioideae is the most ancient subfamily [16], then it was subdivided into the Ixoroideae and finally into the Cinchonoideae.
